# Cachd1 interacts with Wnt receptors and regulates neuronal asymmetry in the zebrafish brain

**DOI:** 10.1126/science.ade6970

**Published:** 2024-05-02

**Authors:** Gareth T. Powell, Ana Faro, Yuguang Zhao, Heather Stickney, Laura Novellasdemunt, Pedro Henriques, Gaia Gestri, Esther Redhouse White, Jingshan Ren, Weixian Lu, Rodrigo M. Young, Thomas A. Hawkins, Florencia Cavodeassi, Quenten Schwarz, Elena Dreosti, David W. Raible, Vivian S. W. Li, Gavin J. Wright, E. Yvonne Jones, Stephen W. Wilson

**Affiliations:** 1 Cell and Developmental Biology, University College London; London, WC1E 6BT, UK; 2 Wellcome Trust Sanger Institute; Cambridge CB10 1SA, UK; 3 Division of Structural Biology, Wellcome Centre for Human Genetics, University of Oxford; Oxford, OX3 7BN, UK; 4 Departments of Otolaryngology-HNS and Biological Structure, University of Washington; Seattle, WA 98195-7420, USA; 5 Ambry Genetics; Aliso Viejo, CA 92656, USA; 6 The Francis Crick Institute; London, NW1 1AT, UK; 7 Institute for Research in Biomedicine (IRB Barcelona), The Barcelona Institute of Science and Technology; 08028, Barcelona, Spain; 8 Institute of Ophthalmology, University College London; London, EC1V 9EL, UK; 9 Center for Integrative Biology, Facultad de Ciencias, Universidad Mayor; Camino La Piramide 5750, 8580745, Santiago, Chile; 10 St. George’s, University of London; London, SW17 0RE, UK; 11 Department of Biology, Hull York Medical School, York Biomedical Research Institute, University of York; York, YO10 5DD, UK

## Abstract

Neurons on left and right sides of the nervous system often show asymmetric properties, but how such differences arise is poorly understood. Genetic screening in zebrafish revealed that loss-of-function of the transmembrane protein Cachd1 resulted in right-sided habenula neurons adopting left-sided identity. Cachd1 is expressed in neuronal progenitors, functions downstream of asymmetric environmental signals, and influences timing of the normally asymmetric patterns of neurogenesis. Biochemical and structural analyses demonstrated that Cachd1 can bind simultaneously to Lrp6 and Frizzled family Wnt co-receptors. Consistent with this, *lrp6* mutant zebrafish lose asymmetry in the habenulae, and epistasis experiments support a role for Cachd1 in modulating Wnt pathway activity in the brain. These studies identify Cachd1 as a conserved Wnt receptor-interacting protein that regulates lateralized neuronal identity in the zebrafish brain.

The nervous systems of bilaterian animals are left-right (LR) asymmetric with respect to neuroanatomy, processing of information, and control of behavior (1–5). Within vertebrates, the epithalamus shows evolutionarily conserved LR asymmetries (6, 7). In zebrafish, the epithalamic dorsal habenulae (dHb) comprise a medial (dHb_M_) domain that is larger on the right and a lateral (dHb_L_) domain that is larger on the left (8–10). Afferent innervation is also asymmetric, with mitral cells innervating the right dHb and parapineal neurons innervating the left dHb (5, 11, 12). Functional asymmetry mirrors neuroanatomy in young fish with, for example, light activating predominantly left-sided dHb_L_ neurons and odor activating a higher proportion of right-sided dHb_M_ neurons (13, 14).

Development of epithalamic asymmetry is dependent on sequential interactions between cell groups that coordinate lateralization of circuit components (15–17). Genetic analyses in zebrafish have revealed roles for Wnt signaling in this process. For example, fish with compromised function of the scaffolding protein Axin1 have symmetric habenulae with right-sided character (18) whereas habenulae are symmetric with left-sided character in fish lacking function of the Tcf7l2 transcriptional effector (19). Wnt signaling also affects the balance between proliferation and neurogenesis (20, 21) suggesting complex regulation of pathway activity during epithalamic development. More generally, Wnt signaling is involved in a wide array of biological processes during embryonic development, throughout life, and in many disease states (22–25). Through studying the role of Wnt signaling in the establishment of brain asymmetry, we identified Cachd1 as a transmembrane component of this highly conserved and multifunctional signaling pathway.

## *rorschach^u761^* mutants show symmetric habenulae owing to a lesion in *cachd1*

**Fig. 1:**
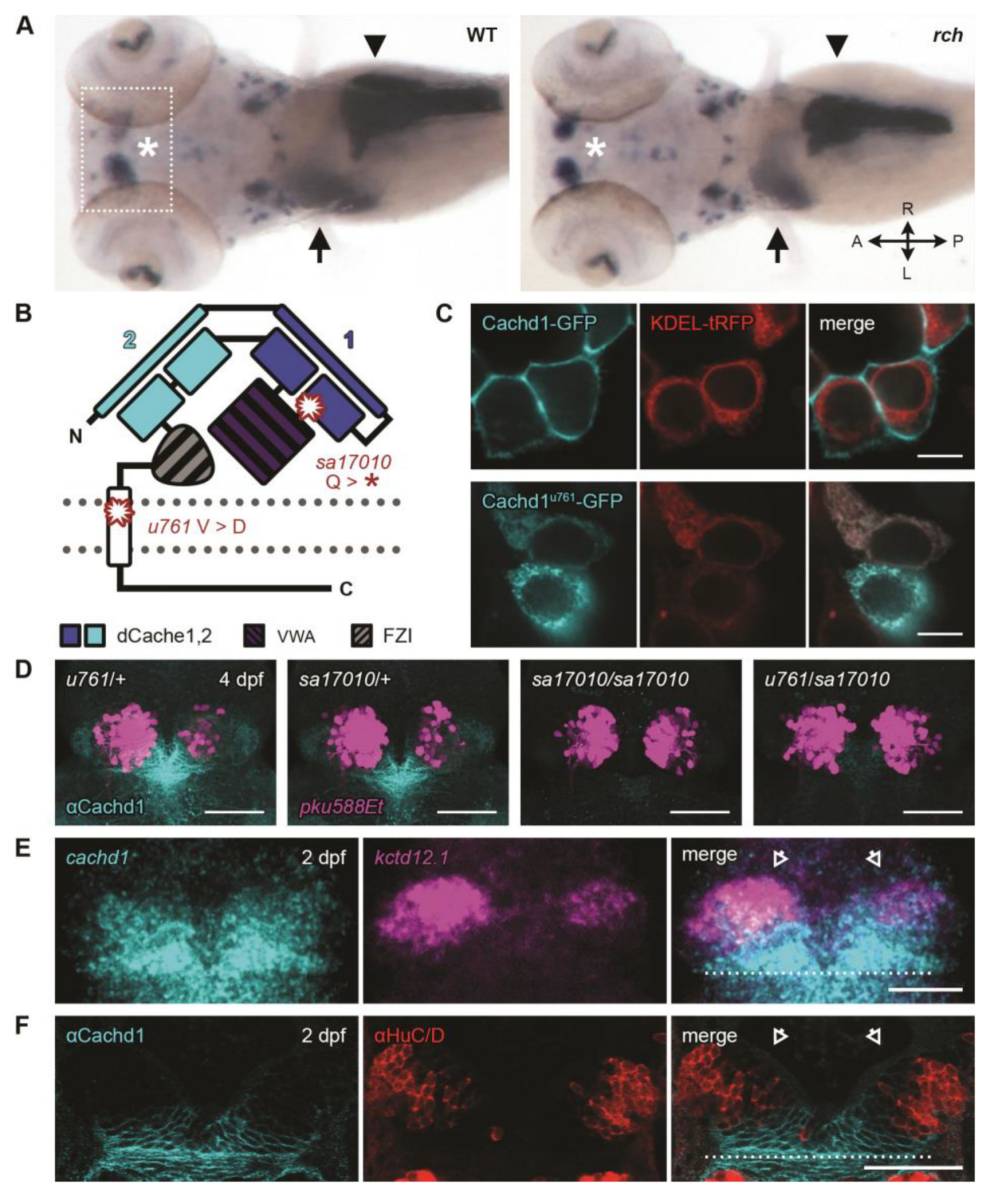
*cachd1* mutants show bilaterally symmetrical, double-left habenulae. (**A**) Dorsal views of whole-mount 5 dpf wild-type sibling and *rorschach* (*rch*/*u761*) mutant larvae showing expression of an asymmetric habenular marker (*kctd12*.*1*, indicated with asterisk; box indicates approximate epithalamic region) and markers for liver (*selenop2*, indicated with arrow), pancreas (*prss1*, indicated with arrowhead), and ventral retina (*aldh1a3*). (**B**) Schematic of Cachd1 protein: two dCache domains (cyan and dark blue), a VWA domain (purple stripes), a FZD interaction domain (FZI; gray stripes), a transmembrane domain (white), and an unstructured cytoplasmic tail. Residues affected in *sa17010* and *u761* alleles are marked in red at approximate positions in primary sequence. (**C**) Fluorescence images of transfected HEK293T cells expressing constructs encoding EGFP-tagged wild-type (top; cyan) or *rch*/*u761* mutant Cachd1 (bottom; cyan) and KDEL-tRFP (red) to mark the endoplasmic reticulum. Scale bar, 10 μm. (**D**) Dorsal views of brains of dissected 4 dpf transgenic siblings from a complementation cross of *sa17010* and *u761* alleles, stained with antibody to Cachd1 (cyan). The *Et(gata2a:EGFP)pku588* (*pku588Et*) transgene is expressed in dHb_L_ neurons (magenta). (**E**) Dorsal view of 2 dpf habenulae after double fluorescent in situ hybridization with *cachd1* antisense riboprobe (cyan) and the dHb_L_ marker *kctd12*.*1* (magenta). (**F**) Dorsal views of 2 dpf habenulae after immunohistochemistry with antibody to Cachd1 (cyan) co-stained with antibody to HuC/D to mark differentiating neurons (red). The dotted lines in (E) and (F) indicate the approximate position of the posterior commissure; open arrowheads indicate the dorsal habenulae. Shown are maximum projections of [(D) and (E)] confocal z-stacks or (F) single confocal slice. Scale bars, 50 μm.

To identify genes potentially involved in establishing brain asymmetry, we screened zebrafish embryos for *N*-ethyl-*N*-nitrosourea (ENU)-induced mutations (19) that alter asymmetric habenular expression of *kctd12*.*1* (8) and identified the homozygous viable *rorschach*^*u761*^ mutant (*rch*). In 4 dpf (days post fertilization) mutant larvae, *kctd12*.*1* expression in the right habenula was increased, reaching a similar degree as on the left, suggesting that both habenulae exhibit left-sided character (Fig. 1A). Other than this fully penetrant habenular phenotype, *rch* mutants were morphologically indistinguishable from wildtypes with normal asymmetry of the viscera.

Mapping placed the *rch* mutation in a 0.28 Mb interval on chromosome 6, and sequencing identified a nonsynonymous single-base pair change in *cachd1* that switched a nonpolar valine to an acidic aspartic acid (V1122D). *cachd1* encodes a 1290 amino acid type I transmembrane protein with dCache and von-Willebrand factor type A (VWA) domains; the V1122D missense mutation occurs within the transmembrane domain (Fig. 1B) and disrupts membrane localization of the protein (Fig. 1C and fig.S1). Embryos homozygous for a likely null mutation in *cachd1* (*sa17010*), producing no detectable Cachd1 protein (fig. S1 and table S1), showed the same habenular double left-phenotype, as did transheterozygote *cachd1*^*u761*^/*cachd1*^*sa17010*^ mutants (Fig. 1D and fig. S2) and embryos injected with splice-blocking *cachd1* morpholinos (fig. S3). Habenular asymmetry was partially restored in homozygous *cachd1*^*u761*^ mutants expressing exogenous Cachd1 from a heat shock promoter during the period of habenular neurogenesis [*Tg(HSE:cachd1, EGFP)w160*] (fig. S4). By contrast, expressing Cachd1-enhanced green fluorescent protein (EGFP) in postmitotic neurons did not rescue the *rorschach* phenotype [*Tg(neurod1:cachd1-EGFP)w162*] (fig. S4). These results show that loss of Cachd1 function during habenular neurogenesis is responsible for the symmetric habenular phenotype.

### Cachd1 is expressed in neuroepithelial cells along the dorsal midline of the brain

To determine the spatiotemporal pattern of *cachd1* expression, we performed colorimetric (fig. S5) and double fluorescent in situ hybridization using epithalamic and habenula markers (Fig. 1E and fig. S6) and immunohistochemistry using an antibody raised against the extracellular domain of zebrafish Cachd1 (Fig. 1F and fig. S1). Before neuronal differentiation, *cachd1* is expressed broadly within the dorsal diencephalon colocalizing with *dbx1b*, a marker of habenula neuron precursors (fig. S6) (26). During the period of habenular neurogenesis (27, 28), *cachd1*/Cachd1 expression becomes restricted to a proliferative neuroepithelial domain adjacent to mature habenula neurons (fig. S7). Although *cachd1* mutants only show an overt mutant phenotype on the right side of the brain, we could not detect obvious asymmetry in *cachd1*/Cachd1 expression until long after habenula asymmetry had been established (fig. S8). Early Nodal signaling-dependent brain (28, 29) and visceral (30) asymmetries were unperturbed in *cachd1* mutant embryos (fig. S9). These results suggest that *cachd1* functions locally within the progenitor domain that gives rise to habenula neurons.

### Cachd1 functions in both habenulae to promote right-sided and/or suppress left-sided character

**Fig. 2:**
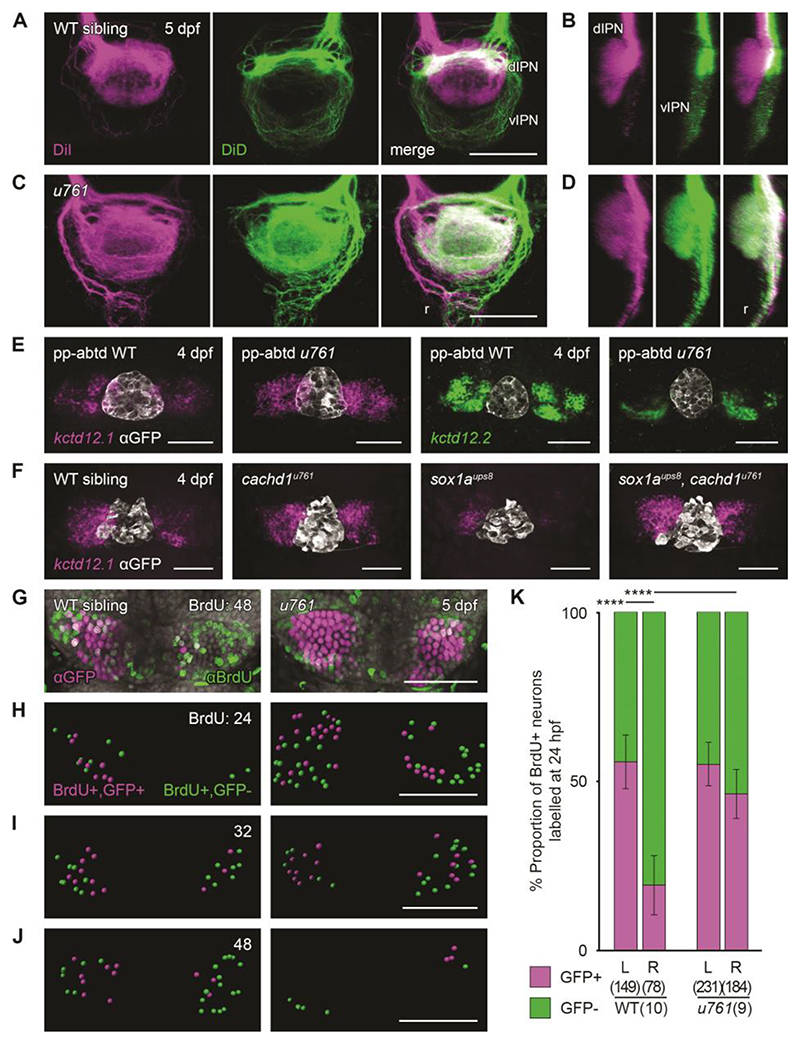
Loss of function of *cachd1* disrupts habenular efferent connectivity, is epistatic to removal of the parapineal signal, and causes precocious neurogenesis. (**A** and **C**) Dorsal views and (B and D) sagittal projections (dorsal left) of the IPN showing DiI (magenta) and DiD (green) labelling of left- and right-sided habenula neuron axon terminals predominantly innervating the dIPN and vIPN respectively, and raphe (r), in 5 dpf wild-type [(A) and (B), *n* = 3] or *cachd1*^*u761*^ mutant [(C) and (D), *n* = 8] larvae. (**E**) Dorsal views of 4 dpf wild-type or *cachd1*^*u761*^ mutant epithalami in which the parapineal was ablated before leftward migration (pineal complex marked by *zf104Tg, u711Tg* alleles with antibody to GFP; white) after double FISH with *kctd12*.*1* (magenta; *n* = 26 of 29 wild-type siblings, 11 of 12 *cachd1*^*u761*^ mutants) or *kctd12*.*2* (green; *n* = 19 of 23 wild-type siblings, 5 of 5 *cachd1*^*u761*^ mutants). (**F**) Dorsal views of 4 dpf larvae from a cross of carriers of *cachd1*^*u761*^ and *sox1a*^*ups8*^ alleles after FISH with *kctd12*.*1* [magenta; pineal complex as (C), white]. *n* = 4 wild-types, 3 *cachd1*^*u761*^ mutants, 4 *sox1a*^*ups8*^ mutants, 3 *sox1a*^*ups8*^, *cachd1*^*u761*^ double mutants. (**G**) Dorsal views of *Et(gata2a:EGFP)pku588* wild-type or *cachd1*^*u761*^ mutant habenulae incubated at 48 hours post fertilization (hpf) with a pulse of BrdU to label newly born neurons, then processed for immunohistochemistry at 5 dpf with antibody to GFP (magenta) and antibody to BrdU (green). DAPI (4′,6-diamidino-2-phenylindole) counterstain marks nuclei (gray). (**H** to **J**) Segmentation of confocal stacks from *Et(gata2a:EGFP)pku588* wild-type or *cachd1*^*u761*^ mutant larvae incubated at (H) 24, (I) 32 and (J) 48 hpf with a pulse of BrdU, then processed at 5 dpf as in (G). Double-positive cells are represented in magenta; BrdU-positive only cells are indicated in green. Times of pulse indicated at top right. (**K**) Quantification of the proportion of BrdU-positive neurons that also expressed *Et(gata2a:EGFP)pku588* (magenta) in 5 dpf wild-type or *cachd1*^*u761*^ larvae incubated with a pulse of BrdU at 24 hpf (all timepoints presented in fig. S11). Error bars represent 95% confidence intervals. Total number of cells and larvae for each genotype indicated in axis label in parentheses. Q′ test of equality of proportions [24 hpf, degrees of freedom (DF) = 3, χ^2^ = 40.94, *P* = 6.7 × 10^-9^], post hoc pairwise comparisons using a modified Marascuilo procedure with Benjamini-Hochberg correction for multiple testing, **** *P* ≤ 0.005. Scale bars, 50 µm in (A) to (H).

Asymmetries in dHb gene expression, synaptic neuropil and targeting of neuronal connections (5, 8–10, 31) were all reduced in *cachd1* mutants so that the right habenula closely resembled the left (Fig. 2, A to D, and fig. S10). The dHb contain two major subtypes of projection neuron present in different frequencies on right and left (9, 10, 31). On the left, dHb_L_ neurons projecting to the dorsal interpeduncular nucleus (dIPN) predominate, whereas on the right, dHb_M_ neurons projecting to the ventral IPN (vIPN) are predominant. Unlike in wild-types, in *cachd1*^*u761*^ mutants the right dHb extensively innervated the dIPN, which is consistent with a higher proportion of right-sided dHb neurons adopting dHb_L_ character (Fig. 2, A to D). These results show that on the right side of the brain, Cachd1 promotes dHb_M_ and/or suppresses dHb_L_ character, but the results do not reveal whether Cachd1 has any function in determining the molecular character of the left habenula.

A small group of parapineal cells is critical for the elaboration of most aspects of left-sided habenula character (5, 8, 10, 32). Consequently, if the parapineal is ablated (Fig. 2E) or fails to signal (Fig. 2F, *sox1a*^*ups8*^ mutant), the left dHb develops with right-sided character. To examine whether the left-sided character of the habenulae in *cachd1* mutants is dependent on parapineal signaling, we ablated the parapineal in *cachd1*^*u761*^ mutants. As expected, ablation in wild-type siblings led to reduced expression *kctd12*.*1*, which is normally high on the left (Fig. 2E), and increased expression of *kctd12*.*2*, which is normally low on the left (Fig. 2E). By contrast, the double-left habenular phenotype of *cachd1* mutants was unaffected by parapineal ablation (Fig. 2E). Similarly, in *cachd1*^*u761*^, *sox1a*^*ups8*^ double mutants, the *cachd1* mutant phenotype was epistatic to the *sox1a* mutant phenotype (Fig. 2F). These results imply that Cachd1 can function on both sides of the brain to suppress left-sided character and/or promote right-sided character. As a corollary to this, it also implies that the signaling role of the parapineal is to antagonize the function of Cachd1 within the left habenula.

Both timing of neurogenesis and the environment into which habenula neurons are born influence their subtype identity (19, 27). Previous work has shown that dHb_L_ neurons tend to be generated earlier than dHb_M_ neurons, and habenular neurogenesis is initiated earlier on the left than on the right (27, 28). Furthermore, early-born neurons on the left have a higher probability of adopting dHb_L_ character than those on the right (19, 28). To elucidate how Cachd1 affects asymmetries in neurogenesis, we performed birth dating experiments to assess both the extent of habenular neurogenesis and timing of birth of *Et(gata2a:EGFP)pku588*-expressing dHb_L_ neurons (*pku588Et*) (Fig. 2, G to K). Neurogenesis began earlier in *cachd1*^*u761*^ mutants compared to wild-types, was symmetric on left and right (Fig. 2, H and I, and fig. S11) and diminished over time (Fig. 2J and fig. S11). In addition, early-born neurons in the right habenula of *cachd1* mutants had a higher likelihood of taking on dHb_L_ character than in wild-types (Fig. 2K and figs. S11 and S12). Cell transplantation experiments showed that as expected for a protein expressed in dividing cells, Cachd1 does not have strictly cell-autonomous consequences on selection of subtype identity (fig. S13).

### Cachd1 binds to Wnt pathway receptors

**Fig. 3:**
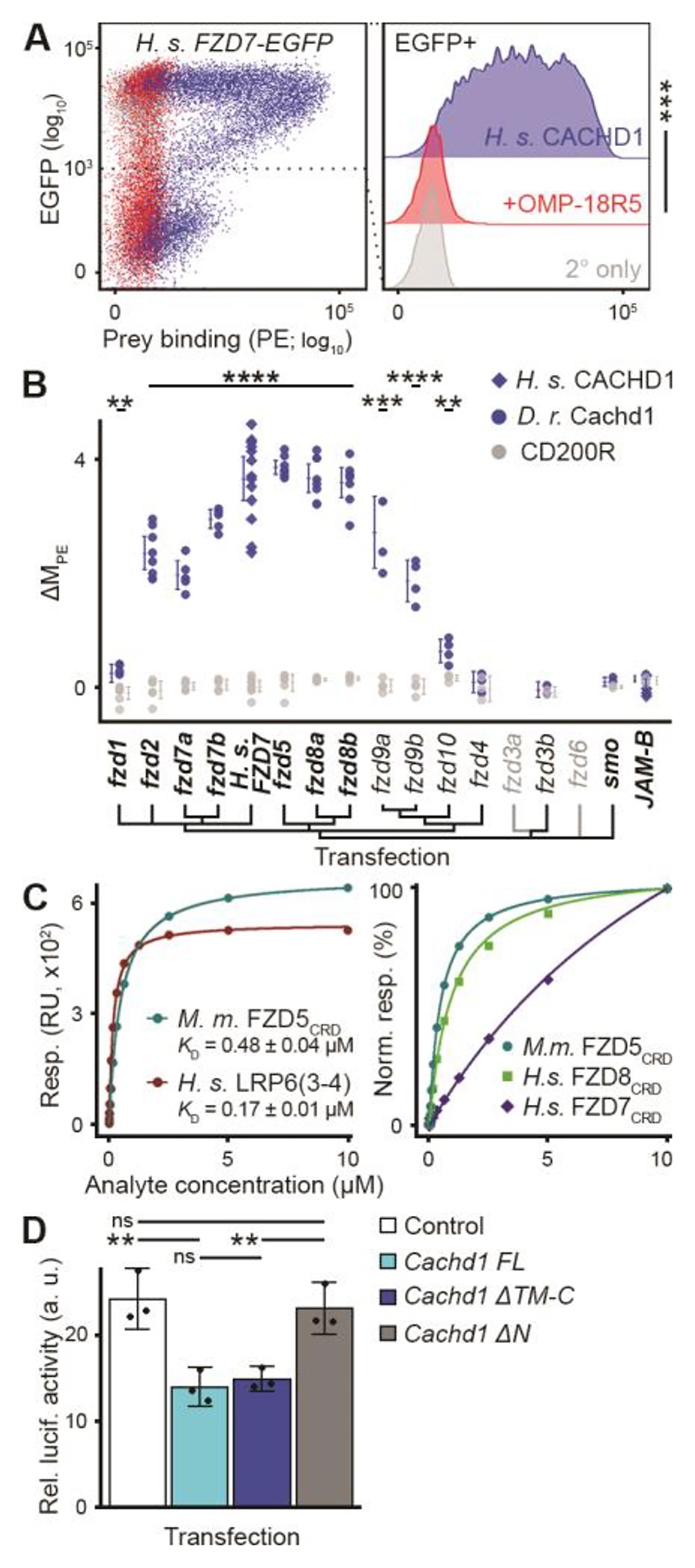
CACHD1 physically interacts with Wnt receptors LRP6 and FZD family members. (**A**) (Left) Representative scatter plot of flow cytometry testing binding of FLAG-tagged CACHD1 prey protein to human FZD7-EGFP transiently transfected HEK293E cells detected by means of phycoerythrin (PE)-conjugated secondary antibody. (Right) Without (blue) or with (red) preincubation with antibody to Frizzled OMP-18R5; secondary only negative control (gray). *n* = 3; one-tailed paired *t* test (DF = 2, *t* = 9.53, *** *P* = 0.0054). (**B**) Dot plot of human (blue diamonds) or zebrafish (blue circles) Cachd1 or negative control CD200R (gray) prey protein binding (standardized as ΔM_PE_ (supplementary materials, materials and methods) to cells transiently transfected with EGFP fusion protein constructs indicated (transfections verified by means of antibody labeling are indicated in bold). Each dot indicates a single experiment; horizontal bars indicate the mean, and error bars indicate 95% confidence intervals. One way Welch test of means (Cachd1 prey versus CD200R prey, not assuming equal variances; *F* = 132.32, DF_num_ = 30.00, DF_denom_ = 34.67, *P* = 5.09 × 10^-28^), post hoc pairwise *t* tests with non-pooled standard deviations, Benjamini-Hochberg correction for multiple testing. Only statistically significant differences between Cachd1 and CD200R prey for individual transfections are presented here for clarity; ** 0.05 ≥ *P* > 0.01, *** 0.01 ≥ *P* > 0.005, **** *P* ≤ 0.005. (**C**) SPR-based determination of *K*_*D*_ (left) for mouse CACHD1_ECD_ analyte binding to immobilized mouse FZD5_CRD_ (cyan) or human LRP6_P3E3P4E4_ (3-4, red), and normalized response curves (right) for different CACHD1_ECD_:FZD_CRD_ interactions. RU, response units. (**D**) TOPFlash responses of HEK293 cells to WNT3A treatment after transfection with a control plasmid (white) or plasmids containing full length rodent *Cachd1* (cyan), *Cachd1* extracellular domain only (blue; *ΔTM-C*), or *Cachd1* transmembrane and intracellular domains only (gray; *ΔN*). Mean responses are shown (*n* = 3 experiments; black dots indicate mean of quadruple technical replicates in each experiment); error bars idicate 95% confidence intervals. One way Welch test of means (not assuming equal variances; *F* = 13.202, DF_num_ = 3.00, DF_denom_ = 4.19, *P* = 0.014), post hoc pairwise *t* tests with non-pooled standard deviations, Benjamini-Hochberg correction for multiple testing. ns, *P* > 0.1, ** 0.05 ≥ *P* > 0.01.

Given that the biochemical function of Cachd1 was unknown, we performed an unbiased screen to find partners that could interact with the extracellular domain of Cachd1. We identified FZD7 as a potential binding partner in a Retrogenix Cell Microarray Technology screen using a human CACHD1 ectodomain (ECD) multimer as prey protein (fig. S14). To validate the interaction, we tested binding of FLAG-tagged CACHD1 to live, intact human embryonic kidney (HEK) 293E cells expressing full-length, EGFP-tagged FZD7 (FZD7-EGFP) by means of flow cytometry. We observed a strong shift of anti-FLAG phycoerythrin-conjugate (PE) fluorescence in EGFP-positive cells tested with CACHD1 prey, but not an unrelated prey protein (Fig. 3, A and B, and fig. S14). Binding was greatly reduced by preincubation with OMP-18R5, a monoclonal antibody to human FZD7 that binds an epitope in the extracellular, N-terminal cysteine-rich-domain (CRD) of several related FZD receptors (Fig. 3A and fig. S15) (33). This suggests that the N-terminal domain of FZD7 contains the binding site for CACHD1.

Because the CRD is very similar between Fzd proteins, using flow cytometry we tested most zebrafish Frizzled family members for binding to Cachd1 (Fig. 3B and fig. S14). Cachd1 prey bound to cells transfected with EGFP fusion constructs of both zebrafish Fzd7 orthologues and most other Frizzled family members tested. Interactions with Fzd1, Fzd2, Fzd7a and Fzd7b were also effectively inhibited by pre-incubation with OMP-18R5 (fig. S15). Furthermore, human CACHD1 prey protein was able to bind zebrafish Frizzled proteins and vice versa (fig. S14), suggesting strong conservation of interactions.

We used surface plasmon resonance (SPR) to measure binding affinity between purified recombinant mammalian CACHD1 and FZD orthologues. Purified mouse CACHD1 extracellular domain analyte (CACHD1_ECD_) interacted with immobilized human FZD7_CRD_, albeit with low affinity [equilibrium dissociation constant (*K*_*D*_) = 14.17 ± 2.18 µM] (Fig. 3C and fig. S16), and with mouse FZD5_CRD_ and human FZD8_CRD_ with much higher affinity (*K*_*D*_ = 0.48 ± 0.04 µM and 0.95 ± 0.06 µM respectively) (Fig. 3C and fig. S16).

Wnt ligands use FZDs and LRP5/6 receptors to initiate Wnt signaling (23). To test whether CACHD1 could also interact with LRP6, we used immobilized human, membrane distal (LRP6_P1E1P2E2_) and membrane proximal (LRP6_P3E3P4E4_) fragments in SPR. CACHD1_ECD_ interacted with high affinity with the LRP6_P3E3P4E4_ fragment (*K*_*D*_ = 0.17 ± 0.01 µM) (Fig. 3C and fig. S16) and with low affinity with LRP6_P1E1P2E2_ (*K*_*D*_ = 5.86 ± 0.62 µM) (fig. S16).

To test whether binding of CACHD1 to canonical Wnt receptors affected signaling, we performed TOPFlash assays in HEK293 cells (Fig. 3D) (34). The response to WNT3A treatment was reduced in cells transfected with full-length *Cachd1* or its ectodomain, but not with the intracellular domain. Furthermore, sensitivity of HEK293 cells to Wnt ligand in the presence of RSPONDIN1 (35) was reduced approximately 89% in cells transfected with *cachd1* (fig. S17). The effect of *cachd1* transfection on canonical Wnt signaling differed between colorectal cancer cell lines, suggesting biological context-dependent regulation of Wnt signaling (fig. S17).

### Structural characterization of CACHD1 complex with FZD5 and LRP6

**Fig. 4:**
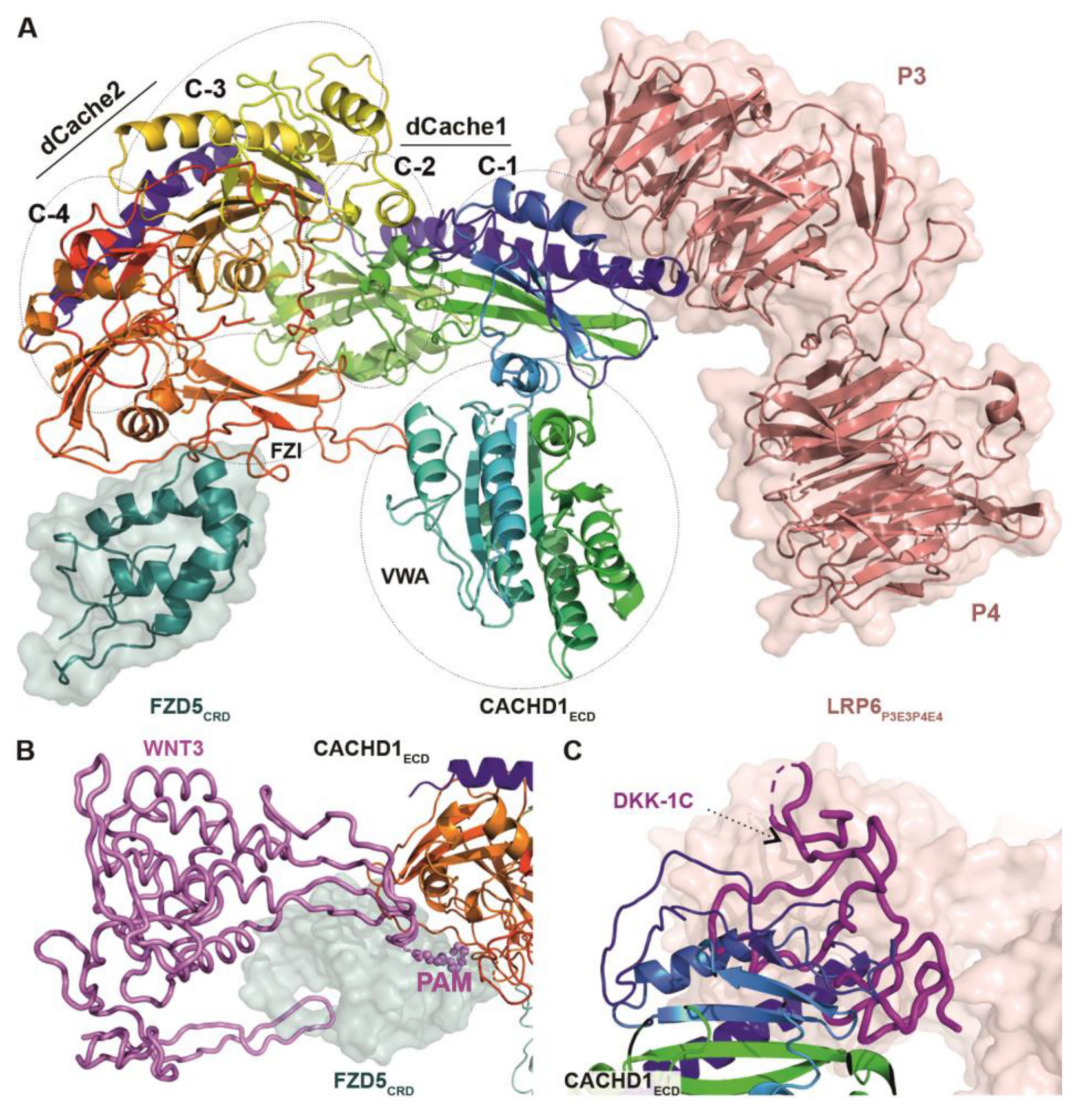
CACHD1 forms a ternary complex with FZD5 and LRP6. (**A**) Cartoon representation of mouse CACHD1_ECD_, [rainbow-colored from N terminus (blue) to C terminus (red)] in complex with mouse FZD5_CRD_ (cartoon and surface in teal) and human LRP6_P3E3P4E4_ (cartoon and surface in salmon pink). The position of the four cache (C-1, -2, -3 and -4), VWA and FZD interaction (FZI) domains of CACHD1_ECD_ are indicated (PDB ID 8S7C). (**B**) Superimposed structures of the FZD8:WNT3 complex (PDB ID 6AHY) with the FZD5_CRD_:CACHD1_ECD_ complex. WNT3 is shown as a violet cartoon tube with palmitoleic acid (PAM) as spheres. (**C**) Superimposed structures of the LRP6:DKK1-C complex (PDB ID 5FWW) with the CACHD1_ECD_:LRP6_P3E3P4E4_ complex. DKK1-C is shown as a magenta cartoon tube.

Guided by our in vitro measurements, we attempted cocrystallization of CACHD1_ECD_ with FZD5_CRD_ and LRP6_P3E3P4E4_. Resultant crystals diffracted to 4.7Å resolution. The structure was determined with molecular replacement by using crystal structures of the CACHD1_ECD_:FZD5_CRD_ complex, previously determined in our laboratory [Protein Data Bank (PDB) ID 9EQ6], and LRP6_P3E3P4E4_ (PDB ID 4A0P) (36). There are three ternary complexes in an asymmetric unit (ASU). Refinement yielded complete structures of equivalent quality for all three copies (table S2), of which one representative complex is depicted in Fig. 4A (PDB ID 8S7C). As expected, CACHD1_ECD_ shows overall structural similarity to the α2δ1 auxiliary subunits of the voltage-gated Ca^2+^ channel Cav1.1 (PDB ID 5GJV; 778 Cα aligned at root mean square deviation = 4.4 Å) (37–39), which contain two dCache domains and a VWA domain. However, the CACHD1 structure reveals an addition to the C-terminal region of the ECD that does not show any homology to known structures in PDB by Dali search (40). This region interfaces with FZD5_CRD_ (Fig. 4A) and we therefore term it the FZD interaction (FZI) domain. The two α helices of the N-terminal dCache domain (C-1) interact with the LRP6_P3_ propeller (Fig. 4A). Thus, CACHD1 serves as a cross-linking component in the ternary complex, independently binding to FZD5_CRD_ and LRP6_P3E3P4E4_.

Structural superpositions show that the CACHD1 binding site on FZD5_CRD_ overlaps with the “thumb” and palmitoleic acid (PAM) lipid binding site (41, 42) required for the receptor-ligand interaction with Wnt (Fig. 4B). Functional studies have indicated that LRP6_P3E3P4E4_ harbors the primary binding site for WNT3A (43) and also for the C-terminal domain of DKK-1 (DKK-1C), an inhibitor that competes with Wnts for binding to LRP5/6 (23). Crystal structures of LRP6_P3E3P4E4_:DKK-1 complexes (PDB IDs 3S2K, 3S8V and 5FWW) detail the interaction of the DKK-1 C-terminal domain with LRP6_P3_ (44–46). Superposition of our LRP6_P3E3P4E4_:CACHD1_ECD_ structure with the LRP6_P3E3P4E4_:DKK-1C complex (PDB ID 5FWW) shows a steric clash between the CACHD1 C-1 helices and DKK-1C (Fig. 4C). This suggests that CACHD1 may also compete with WNT3A for binding to the LRP6_P3_ propeller (43). These biophysical and structural analyses suggest that CACHD1 binds members both of the FZD family and the LRP6 Wnt co-receptors.

### *cachd1* genetically interacts with Wnt pathway genes

**Fig. 5:**
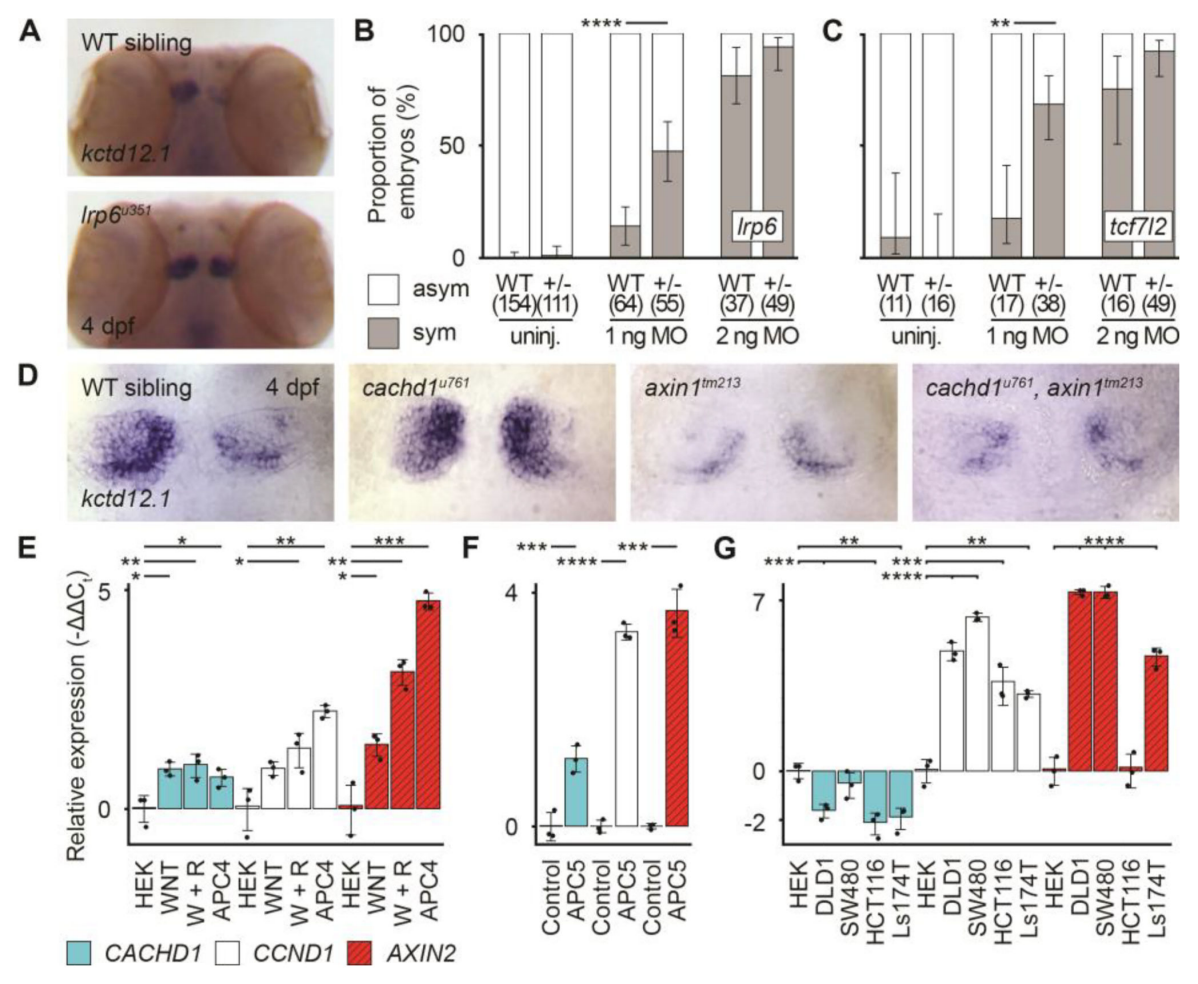
*cachd1* interacts genetically with Wnt pathway components. (**A**) Dorsal views of wholemount 4 dpf wild-type sibling (*n* = 12) and *lrp6*^*u351*^ mutant (*n* = 9) heads stained for expression of *kctd12*.*1*. (**B** and **C**) Graphs showing the percentage of (B) 4 dpf wild-type siblings and *lrp6*^*u349*/+^ larvae or (C) wild-type siblings and *tcf7l2*^*zf55/+*^ larvae with (gray; sym) or without (white; asym) a symmetric bilateral left phenotype in uninjected larvae and larvae injected with a suboptimal (1 ng) or standard dose (2 ng) of *cachd1* morpholino (MO1). Error bars indicate 95% confidence intervals of the proportion. Q′ test of equality of proportions [(B) DF = 2, χ^2^ = 18.71, *P* = 8.66 × 10^-5^; (C) DF = 2, χ^2^ = 7.93, *P* = 0.019) and post hoc modified Marascuilo procedure with Benjamini-Hochberg correction for multiple testing. (**D**) Dorsal views of the habenulae of wholemount 4 dpf larvae from an incross of *cachd1*^*u761*^ and *axin1*^*tm213*^ mutants, showing expression of *kctd12*.*1. n* = 5 wild-types, 6 *cachd1*^*u761*^ mutants, 3 *axin1*^*tm213*^ mutants and 3 *cachd1*^*u761*^, *axin1*^*tm213*^ double mutants. (**E** to **G**) Quantitative RT-PCR data showing relative expression (-ΔΔ*C*_t_ values) of *CACHD1* and known Wnt-responsive genes (*CCND1, AXIN2*) in (E) HEK293 (HEK) cells untreated, incubated with WNT3A alone (WNT) or WNT3A and RSPONDIN1-conditioned media (W + R), or stable *APC* mutant cells (*APC4*); (F) wild-type (Control) and *Apc* mutant (*APC5*) mouse organoids; and (G) colorectal cancer-derived cell lines with mutations in Wnt pathway genes (*APC* mutants, DLD1, SW480; *CTNNB1* mutants, HCT116, Ls174T). Data is presented as mean -ΔΔ*C*_t_ values compared to expression of *ACTB* (human) or *Hrpt1* (mouse) reference genes and untreated controls (HEK293 cells or wild-type organoid). Individual points indicate biological replicates (each an average of three technical replicates), *n* = 3; error bars indicate 95% confidence intervals. One way Welch test of means [not assuming equal variances; (A) *F* = 58.83, DF_num_ = 11.00, DF_denom_ = 9.41, *P* = 3.03 × 10^-7^; (B) *F* = 225.66, DF_num_ = 5.00, DF_denom_ = 5.16, *P* = 5.12 × 10^-6^; (C) *F* = 236.49, DF_num_ = 14.00, DF_denom_ = 11.33, *P* = 7.67 × 10^-12^], post hoc pairwise *t* tests with non-pooled standard deviations and Benjamini-Hochberg correction for multiple testing; only statistically significant differences with control samples (HEK or Control) are presented here for clarity. * 0.1 ≥ *P* > 0.05, ** 0.05 ≥ *P* > 0.01, *** 0.01 ≥ *P* > 0.005, **** *P* ≤ 0.005.

If Cachd1 functions with Fzd and Lrp6 proteins during habenular development, then abrogation of Fzd and/or Lrp6 function may also result in habenular asymmetry phenotypes. The Fzd family is large (23), so we focused analysis on Lrp6 function in habenular development. We generated several predicted *lrp6* null alleles and found that homozygous mutants showed a fully penetrant, symmetrical double-left habenular phenotype (Fig. 5A, fig. S18 and table S3). We tested for a genetic interaction between *cachd1* and *lrp6* by injecting a *cachd1* splice-blocking morpholino into heterozygous *lrp6*^*u349*/+^ embryos at a low dose that rarely leads to symmetric habenulae in wild-types. We observed that heterozygous *lrp6*^*u349/*+^ larvae were approximately three times more likely to show bilaterally symmetric habenular *kctd12*.*1* expression than wildtype siblings (Fig. 5B). Confirming that this difference was not due to morpholino efficacy, injection of a standard dose caused bilateral symmetry in both genotypes (Fig. 5B). Because these results suggest Cachd1 and Lrp6 function in the same developmental pathway, we next assessed genetic interactions between *cachd1* and two other Wnt pathway genes implicated in habenular development: *axin1* and *tcf7l2* (18, 19).

Tcf7l2 is a transcriptional effector of Wnt signaling and loss of *tcf7l2* function results in symmetric habenulae with double-left character (19). *tcf7l2*^*zf55/*+^ heterozygotes show a wild-type habenular phenotype, but when *cachd1* expression was reduced in *tcf7l2*^*zf55/*+^ heterozygotes through injection of low dose *cachd1* morpholino, many larvae showed symmetric, double-left habenulae (Fig. 5C). Consequently, reduced activity of both genes results in a phenotype comparable with that seen when either alone is fully abrogated.

Compromised function of the β-catenin degradation complex scaffolding protein Axin1 results in symmetric habenulae with double-right character (18), in contrast to the phenotype of *cachd1* mutants. *axin1*^*tm213*^, *cachd1*^*u761*^ double mutants exhibited the *axin1* mutant phenotype (as assessed by expression of *kctd12*.*1*) (Fig. 5D). Consequently, compromised Axin1 function is epistatic to loss of Cachd1 function, consistent with Axin1 functioning downstream of Cachd1 and the Fzd/Lrp6 receptor complex.

Wnt signaling often regulates expression of Wnt-pathway genes (23), and the spatially localized expression of *cachd1* along the dorsal forebrain is similar to that of other Wnt pathway genes such as *wnt1, wnt3a, wnt10b* (47), *axin2* and *lef1* (fig. S19). To test whether CACHD1 is itself a target of Wnt signaling, we used quantitative reverse transcription polymerase chain reaction (RT-qPCR) to assess CACHD1 expression in HEK293 cells under different conditions: treatment with WNT3A-conditioned media, treatment with WNT3A+RSPONDIN1-conditioned media, and carrying a stable mutation in *APC* (48). Moreover, we performed the same experiment in mouse *Apc*-mutant organoids. *CACHD1*/*Cachd1* showed similar transcriptional responses to enhanced Wnt pathway activity as other Wnt target genes (Fig. 5, E and F), whereas *CACHD1* expression was reduced in cells derived from colorectal cancers (Fig. 5G). Complementarily, global overexpression of *cachd1* in vivo caused a reduction in expression of the Wnt target *axin2* (fig. S20).

These results provide compelling evidence that the structural interactions we have demonstrated are pertinent to Cachd1 function in the developing brain.

## Discussion

Our studies identify Cachd1 as a component of the Wnt pathway that bridges Fzd and Lrp6 Wnt receptors and plays an important role in the developing zebrafish brain. Recent studies in mice and humans suggest CACHD1 may function in other contexts involving Wnt pathway activity (49, 50). We demonstrate evolutionary conserved interactions between CACHD1 and multiple FZD receptors through a previously unidentified FZI domain that could potentially compete with Wnts binding to FZDs through their PAM moiety. Similarly, binding of the dCache domain of CACHD1 to LRP6 may compete with Wnts and the Wnt inhibitor, DKK-1.

The simultaneous binding of Cachd1 to Fzd and Lrp6 receptors could potentially activate signaling by clustering the cytoplasmic apparatus as observed with artificial ligands (51). This would be consistent with the similarity of habenular phenotypes in *cachd1, lrp6* and *tcf7l2* (19) mutants and contrast the phenotype of *axin1* mutants in which the pathway is overactivated (18). However, in vitro reporter assays show that Cachd1 can inhibit Wnt signaling and we remain circumspect about the consequences of Cachd1 function in the developing habenulae given the complexity of events in vivo.

Our study suggests that asymmetric Cachd1-dependent modulation of Wnt signaling leads to lateralization of habenula neurons by altering both timing of neurogenesis and the probabilistic selection between alternate neuronal fates. We show that Cachd1 is present and can function on both sides of the brain, but its activity on the left is antagonized by an unknown signal (or signals) from the parapineal. During habenular development, as in many other contexts, Wnt signaling functions at multiple stages and in multiple processes, from proliferation to acquisition and maintenance of cell identity [this study and (18–21)]. It is largely unclear how this complexity of pathway activity and outcome is effected, and an attractive possibility is that context-dependent activity of Cachd1 may contribute to this poorly understood aspect of Wnt signaling.

## Supplementary Material

Supplementary Materials

## Data Availability

The crystal structure of the CACHD1_ECD_:FZD5_CRD_:LRP6_P3E3P4E4_ ternary complex is available at the Research Collaboratory for Structural Bioinformatics Protein Data Bank under accession no. 8S7C. Further information and requests relating to zebrafish resources and reagents, including mutants generated in this study, should be directed to G.T.P. (g.powell@ucl.ac.uk) and S.W.W. (s.wilson@ucl.ac.uk), information and requests relating to structural biology and biochemistry should be directed to E.Y.J. (yvonne.jones@strubi.ox.ac.uk) and Y.Z. (yuguang.zhao@strubi.ox.ac.uk).
