# Supplementary material for: Cachd1 interacts with Wnt receptors and regulates neuronal asymmetry in the zebrafish brain
Source: Science. Author manuscript; Available in PMC 2024 May 23. (PMC7615972; doi:10.1126/science.ade6970)
Supplement: Supplementary Materials [file EMS195897-supplement-Supplementary_Materials.pdf]

This is the author's version of the work. It is posted here by permission of the AAAS for personal use, not for redistribution. The definitive version was published in *Science* on 03/05/2024, DOI: 10.1126/science.ade6970.

Supplementary Materials for

Cachd1 interacts with Wnt receptors and regulates neuronal asymmetry in the zebrafish brain

Gareth T. Powell[1,2]†, Ana Faro[1]†, Yuguang Zhao[3]*†, Heather Stickney[1,4,5]†, Laura Novellasdemunt[6,7], Pedro Henriques[1], Gaia Gestri[1], Esther Redhouse White[1], Jingshan Ren[3], Weixian Lu[3], Rodrigo M. Young[1,8,9], Thomas A. Hawkins[1], Florencia Cavodeassi[1,10], Quenten Schwarz[8], Elena Dreosti[1], David W. Raible[4], Vivian S. W. Li[6], Gavin J. Wright[2,11], E. Yvonne Jones[3]*, Stephen W. Wilson[1]*

Corresponding authors: Email: s.wilson@ucl.ac.uk (S. W. W.), yvonne.jones@strubi.ox.ac.uk (E. Y. J.), yuguang.zhao@strubi.ox.ac.uk (Y. Z.)

Supplementary Materials contains:

Author contributions

Materials and Methods

Figs. S1 to S20

Tables S1 to S8

References (*52–90*)

Author contributions:

The senior authors wish to emphasize that all four lead authors made equally important contributions to this study and are happy for individuals to list the joint authors in whichever order they wish on CVs and other documents.

GTP devised and performed experiments (generated/gathered reagents, protein interactions/immunocytochemistry using flow cytometry, immunohistochemistry, *in situ* hybridization chain reaction, *lrp6* mutagenesis, morpholino injections), analyzed data, contributed to writing the paper, prepared figures. AF devised and performed experiments (*in situ* hybridization, immunohistochemistry, BrdU pulse-chase, parapineal ablations, lipophilic dye labelling, epistasis, morpholino injections), analyzed data. YZ devised and performed experiments (structural biology, protein interactions using surface plasmon resonance), analyzed data, prepared figures. HS devised and performed experiments (ENU mutagenesis and screening, genetic mapping, gene identification, morpholino injections, *in situ* hybridization, immunohistochemistry, lipophilic dye labelling, transgenesis and heat shock experiments, tissue culture and transfection), analyzed data. LN devised and performed experiments (qRT-PCR, generated organoids), analyzed data. PH devised and performed experiments (parapineal ablations and *in situ* hybridization), analyzed data. GG designed and performed experiments (phenotype characterization, transplantation). ER-W performed experiments (epistasis and *in situ* hybridization). JR analyzed data (structural phasing/model building). WL generated reagents for research (tissue culture/protein production). RMY, HS, TAH, FC and QS undertook the screen that isolated the *rorschach* mutant. ED generated reagents for research (transgenesis). DWR devised experiments and provided funding for research (supported HS). VWSL provided funding for research (supported LN). GJW

This is the author's version of the work. It is posted here by permission of the AAAS for personal use, not for redistribution. The definitive version was published in *Science* on 03/05/2024, DOI: 10.1126/science.ade6970.

devised experiments and provided funding for research (supported GTP). EYJ provided funding for research (supported YZ, JR, WL), devised experiments, contributed to writing the paper. SWW provided funding for research (supported GTP, AF, HS, PH, ER-W, RMY, TAH, QS, FC, ED), devised experiments, wrote the paper.

Materials and Methods:

Zebrafish husbandry and fish lines

Zebrafish experiments and husbandry followed standard protocols *(52)* in accordance with the UK Home Office (project licenses 70/7071, 70/8449 and PP4300676) or University of Washington Institutional Animal Care and Use Committee guidelines: Office of Laboratory Animal Welfare (OLAW) Assurance Number for the University of Washington D16-00292.

Zebrafish were maintained in designated facilities on a 14h/10h light:dark cycle. Embryos and fry were obtained by natural spawning of wildtype, *cachd1*^{u761} (this study, below), *cachd1*^{sa17010} (Zebrafish Mutation Project; ZFIN: ZDB-ALT-131220-250) *(53)*, *sox1a*^{ups8} (ZDB-FISH-200611-5) *(32)*, *lrp6*^{u348}, *lrp6*^{u349}, *lrp6*^{u350}, *lrp6*^{u351} (this study, below), *axin1*^{tm213} (ZDB-FISH-150901-22104) *(54)*, *tcf7l2*^{zf55} (ZDB-FISH-150901-25729) *(55)*, *Tg(foxd3:GFP)zf104 (56)*, *Tg(-1.6flh:GAP-EGFP)u711* (ZDB-FISH-200530-1) *(5)*, *Et(gata2a:EGFP)pku588* (ZDB-FISH-150901-5098) *(57)*, *Tg(HSE:cachd1, EGFP)w160* (this study, below), *Tg(neurod1:Cachd1-EGFP)w162* (this study, below), *Tg(gng8:EGFP)u775* (this study, below) fish.

Embryos were routinely stored in fish system water supplemented with methylene blue, or E3 embryo medium at 28°C. Where necessary, embryos were treated with 0.2 mM 1-phenyl 2-thiourea (PTU) to prevent pigment formation.

Generation of mutant and transgenic lines

The *u761* mutant was generated by ENU mutagenesis. Mutations were induced in wildtype male AB/TL fish by four rounds of 3 mM ENU treatment as previously described *(58)*.

An allelic series of predicted *lrp6* nonsense mutants (*u348*, *u349*, *u350* and *u351*) were recovered from founders mutated using CRISPR/Cas9. Briefly, *in vitro* transcribed, capped *cas9* mRNA and sgRNAs, prepared as described in *(59)* using T4 DNA polymerase (New England BioLabs, Ipswich, MA, USA) and mMessage mMACHINE (Ambion, Austin, TX, USA), complementary to exon 2 of *lrp6* (sg1: GGCCAACGCCACGCTGGTGA, sg2: GGCCAGACCGGAGATGACGG; Table S4) were microinjected into the cell of 1 cell stage embryos. Injected fish were raised to adulthood and genotyped for mosaicism of exon 2 using high resolution melting analysis (HRMA, see Table S5). Fish with a high degree of mosaicism were prioritized for outbreeding to generate F1s which were subsequently genotyped using headloop PCR combined with Sanger sequencing to identify alleles of interest *(60)* (Tables S3 and S5).

The *Tg(HSE:cachd1, EGFP)w160*, *Tg(neurod1:Cachd1-EGFP)w162* and *Tg(gng8:EGFP)u775* lines were generated by Tol2-mediated mutagenesis. Briefly, 1-cell zebrafish embryos were co-injected with *pTol2 HSE:cachd1, EGFP* (*w160Tg*), *pTol2 neurod1:cachd1-EGFP CG2* (*w162Tg*) or *pTol2 gng8:EGFP* (*u775Tg*) construct (25-50 pg; see below) and capped transposase mRNA (40 pg) and the embryos raised to adulthood. Offspring of the injected, adult fish were screened for germline transmission of the transgene and their progeny raised.

This is the author's version of the work. It is posted here by permission of the AAAS for personal use, not for redistribution. The definitive version was published in *Science* on 03/05/2024, DOI: 10.1126/science.ade6970.

Cloning and genotyping of *u761*

Having used a combination of backgrounds to generate our F2s, we mapped *u761* in F3 embryos. We used bulked segregant analysis *(61)* followed by high resolution SSLP and SNP analyses to localize *u761* to a 0.28 MB interval on LG6 between a SNP in the first coding exon of *ak4* (2/5212 recombinants; *ak4* e1 primers; see Table S5) and an SSLP in intron 8-9 of *cachd1* (1/5212 recombinants; *cachd1* i8-9 primers; see Table S5). Sequencing of *cachd1* cDNA revealed a T to A transversion in the 24th exon of *cachd1* that causes a valine to aspartic acid amino acid substitution in its transmembrane domain (reverse strand 6:31607781 T>A, 1122V>D, Zv11 assembly). Mutants were subsequently genotyped with DCAPs primers (Table S5, *u761*-AloI primers) and the restriction enzyme AloI, which cuts the mutant allele, and then more routinely by KASP assay (see below).

Morpholino knockdown

Two non-overlapping morpholino antisense oligonucleotides for *cachd1* (MO1: GTGTATTTTCCTACCTGCATGGTGA; MO2: AGGGATGATGTCTAACTCACCTGCT) were obtained from GeneTools (Philomath, OR, USA) and microinjected into the yolks of 1-cell stage zebrafish embryos in 1 nL volumes for a total dose between 4 ng and 0.5 ng, depending upon the experiment.

DNA extraction, KASP and HRMA genotyping

Embryos or larvae were lysed at 95ºC in 25 mM KOH, 0.2 mM EDTA for 30 minutes, cooled to 4ºC and briefly vortexed to disrupt remaining tissue. The lysate was briefly spun in a centrifuge to collect, then neutralized with an equal volume of 40 mM Tris-HCl, pH 5.

KASP or HRMA genotyping of DNA lysates was performed as per manufacturer’s instructions, using either 2× KASP Master Mix with standard ROX (LGC Biosearch Technologies, Hoddesdon, UK) or 2× Precision Melt Supermix (Bio-Rad, Hercules, CA, USA), respectively, and a Bio-Rad CFX96 qPCR machine (see Table S5 for primer details).

Melting curves from HRMA genotyping were analyzed using Precision Melt Analysis software (version 1.2; Bio-Rad).

cDNA and plasmid constructs

Total RNA was extracted and purified from pools of 10-20 embryos (wildtype, *rch*, or *cachd1* MO-injected, depending on experiment) with TRIzol reagent (Life Technologies, Grand Island, NY, USA) according to the manufacturer’s instructions. cDNA was then produced using the SuperscriptIII first strand synthesis system for RT-PCR (Life Technologies; see Table S5).

pCS2+ cachd1-EGFP and *pCS2+* $cachd1^{u761}$*-EGFP*: Full-length *cachd1* and $cachd1^{u761}$ were amplified from cDNA with Phusion DNA polymerase (New England BioLabs) and primers tagged with SalI and SacII restriction enzymes sites (see Table S5 for primer sequences). The resulting PCR fragment and the *pEGFP-N1-1* vector were sequentially digested with SalI and SacII prior to ligation with Quick ligase (New England Biolabs) to make a *pcachd1-EGFP-N1-1* plasmid. The *pcachd1-EGFP-N1-1* construct was then cut with SalI and HpaI and the fragment containing *cachd1-EGFP* cloned between the SalI and SnaBI sites of the *pCS2+* vector.

pTol2 neurod1:cachd1-EGFP CG2: Full-length *cachd1-EGFP* was PCR-amplified from *pCS2+ cachd1-EGFP* plasmid with AttB1-tagged primers. The Gateway cloning method was

This is the author's version of the work. It is posted here by permission of the AAAS for personal use, not for redistribution. The definitive version was published in *Science* on 03/05/2024, DOI: 10.1126/science.ade6970.

used to combine this PCR product with *pDONR 221, p5E NeuroD, p3E polyA* and *pDest tol2 CG2* following the manufacturer's protocols.

pTol2 HSE:cachd1, EGFP: Full-length *cachd1* was amplified from cDNA using high-fidelity Phusion DNA polymerase (New England Biolabs) and then phosphorylated with PNK (New England BioLabs; see Table S5 for primers). The phosphorylated fragments were then cloned into the StuI site of a *pCS2+* vector treated with Antarctic Phosphatase (New England Biolabs) to prevent recircularization. The resulting vectors were cut with BamHI and SnaBI and the *cachd1*-containing fragments cloned into the BamHI and EcoRV sites of the *pTol2 HSE:EGFP* vector *(62)* to obtain the *pTol2 HSE:cachd1, EGFP* construct for injection.

pTol2 gng8:EGFP: a 3060 bp promoter region of the *gng8* gene was amplified by PCR (see Table S5 for primer details) and cloned into a *TOPO-TA* vector. This fragment was subcloned into *pEGFP-N1*, upstream of the *EGFP* open reading frame, and the subsequent *gng8:EGFP* fragment cloned into *pTol2*.

For flow cytometry protein production, the coding sequence for the ectodomain of human and zebrafish *CACHD1* (truncated before the transmembrane domain at P1095/P1108 respectively) was codon optimized for HEK cells and synthesized by GeneArt (Thermo Fisher Scientific, Waltham, MA, USA). These fragments had NotI and AscI target sequences at the 5′ and 3′ ends, respectively, for subcloning into prey protein and ectodomain bait protein production vectors.

Human and zebrafish CACHD1 prey protein expression constructs (ectodomain fused to a COMP domain, β-lactamase domain and FLAG tag) and zebrafish Cachd1 ectodomain production constructs (ectodomain fused to hexahistidine and BirA ligase peptide substrate tags) were prepared by NotI/AscI restriction enzyme double digest (New England Biolabs) of *pTT3*-based vector backbones *(63)* (Addgene IDs 71471 and 36153) and shuttle vectors containing the synthesized fragments, followed by ligation with T4 ligase (New England Biolabs). The resulting constructs were screened by Sanger sequencing to confirm correct in-frame insertion.

To create human and zebrafish FZD-EGFP bait protein constructs, IMAGE consortium clones *(64)* (see Table S6 for details) were used as templates in PCR reactions to generate full length inserts (including the seven transmembrane domains) with NotI and AscI target sequences at the 5′ and 3′ ends, respectively, except for *fzd4* and *fzd9a* where the insert was synthesized by GenScript (Piscataway, NJ, USA) as no complete full length clone was available. *fzd1* and *fzd8b* both had NotI/AscI restriction sites in the respective coding sequences, so fusion PCR was used to generate full length inserts with synonymous mutations in the recognition sequences (see Table S5 for primer sequences). The PCR products were purified using a Qiaquick PCR purification kit (Qiagen, Hilden, Germany) and then digested with NotI/AscI (New England Biolabs) and ligated to a *pTT3* vector containing *EGFP* (see below). The resulting constructs were verified by Sanger sequencing.

The *pTT3-EGFP* vector was constructed by replacing the C-terminal tag encoding region of a bait protein vector *(65)* (Addgene ID 36150) with *EGFP*. The bait protein vector was digested with AscI/BamHI (New England Biolabs) to remove the tag encoding region and then ligated to an *EGFP* insert generated by PCR using primers with AscI and BamHI tails (see Table S5 for primer sequences). The resulting vector was verified by Sanger sequencing to ensure in-frame insertion of the EGFP coding sequence.

This is the author's version of the work. It is posted here by permission of the AAAS for personal use, not for redistribution. The definitive version was published in *Science* on 03/05/2024, DOI: 10.1126/science.ade6970.

All constructs used for producing proteins for surface plasmon resonance and crystallography were based on the mammalian stable expression vector *pNeoSec (66)*. Mouse CACHD1 extracellular domain (UniProt: Q6PDJ1, residues D50–S1107) was derived from IMAGE clone 6834428 (Table S6; Source Bioscience). Mouse FZD5 cysteine rich domain (UniProt: Q9EQD0, residues A27-T157), human FZD7 CRD (UniProt: O75084, residues Q33-G170), human FZD8 CRD (UniProt: Q9H461, residues A28-T158) were synthesized (GenScript). Human $LRP6_{P1E1P2E2}$ (UniProt: O75581, residues A20-P630) and $LRP6_{P3E3P4E4}$ (residues V629-G1244) domains were described previously *(51)*.

Tissue culture and cell transfection

HEK293T (ATCC: CRL-3216), APC4 (APC4 line was generated from HEK293T cells by CRISPR targeting *APC* with truncation at 1225 a. a.) *(48)*, SW480 (ATC: CCL-228), Ls174T (ATC: CL-188), DLD1 (ATC: CCL-221) and HCT116 (ATC: CCL-247) were maintained in DMEM GlutaMAX (GIBCO) supplemented with 5% fetal bovine serum (FBS) (GIBCO), 100 U/mL penicillin (GIBCO) and 100 mg/mL streptomycin (GIBCO). All cells were maintained at 37°C in an incubator with 5% CO_2. Cells were seeded in plates 24 hours before transfection, and plasmids were transfected using polyethylenimine (PEI; Polysciences, Warrington, PA, USA) or Fugene 6 (Promega) according to the manufacturer's instructions.

HEK293T cells were simultaneously transfected with 1 µg *pCS2Cachd1-EGFP* or *pCS2Cachd1_V1122D-EGFP* and 1 µg *KDEL-tRFP* plasmid using Fugene 6 transfection reagent (Promega). EGFP/tRFP expression was confirmed 24 hours post transfection and the cells fixed at 42 hours post transfection and imaged on a spinning disk microscope (see below).

For protein production and flow cytometry experiments, suspension cultures of HEK293E *(67)* or HEK293-6E *(68)* cells were transfected using linear PEI:plasmid complexes, incubated for between 2-6 days and then harvested by centrifugation. The resulting cells or conditioned media were then used for downstream experiments *(63)*. Briefly, HEK293E or HEK293-6E cells were maintained in suspension cultures in Freestyle 293 Expression Media (Gibco, Waltham, MA, USA) supplemented with heat-inactivated fetal calf serum (1%) and G418 (geneticin, 50 µg/mL; Sigma-Aldrich, St. Louis, MO, USA), routinely maintained at densities between 2.5×10^5 and 4×10^6 cells/mL in Erlenmeyer flasks (Corning, Corning, NY, USA) in a humidified orbital shaker at 37ºC, 5% CO_2. One day before transfection, cultures were split down to 2.5×10^5 cells/mL in standard media or, in the case of biotinylated protein production, media supplemented with D-Biotin (100 µM). Plasmids for transfection were prepared using PureLink HiPure Plasmid Maxiprep kit, as per manufacturer's instructions (Thermo Fisher Scientific), and resuspended in ddH_2O at 1 mg/mL. For each transfection, purified plasmid was mixed with linear 25 kDa PEI (Polysciences) at a ratio of 1 µg DNA:2.2 µg PEI (per 5×10^6 cells) in unsupplemented Freestyle 293 Expression Media (1/10th culture volume), vortexed and left to stand for 5 minutes at room temperature to allow complexes to form, before mixing into the cell cultures (for example, to transfect a 50 mL culture with density 5×10^5 cells/mL, 50 µL of plasmid was mixed with 110 µL PEI 1 mg/mL in water, in 2 mL media). In the case of biotinylated protein production, cells were co-transfected with an additional secreted BirA ligase plasmid *(63)* (Addgene ID 64395) included in the transfection mixture at a ratio of 10 µg DNA:22 µg PEI: 1 µg BirA. Transfected cultures were incubated for approximately 2 (for flow cytometry) or 6 days (protein production) before harvesting by centrifugation (200 × g or 3200 × g, respectively) to separate cells from conditioned media.

This is the author's version of the work. It is posted here by permission of the AAAS for personal use, not for redistribution. The definitive version was published in *Science* on 03/05/2024, DOI: 10.1126/science.ade6970.

Proteins used in surface plasmon resonance and crystallography were derived from stable cell lines established by G418 selection (1 mg/mL, Sigma) of transfected HEK293S GnTI(−) cells *(69)*.

Organoid culture

Organoids were cultured as described in *(70)*, except that Matrigel was replaced with Cultrex© BME, Type 2 RGF PathClear (Amsbio, Abbingdon, UK, 3533-010-02). Briefly, organoid basal media contains EGF (Invitrogen, Waltham, MA, USA PMG8043), NOGGIN and RSPONDIN1 (ENR). NOGGIN and RSPONDIN1 conditioned media (CM) were generated from HEK293T cells. APC5 organoids were previously generated by CRISPR/Cas9, generating a truncation in APC at 680 aa *(48)*. WNT3A CM was generated from L cells (ATCC: CRL-2647).

TOPFlash assay

HEK293 SuperTopFlash cells (STF, ATCC: CRL-3249) were seeded into a 96-well plate (10^5 cells/well) and transfected with a constitutive Renilla luciferase plasmid (pRL-tk; Promega) together with an expression plasmid of mouse *Cachd1* (ectodomain only, transmembrane with cytoplasmic domain or full length) or a control plasmid (expressing bacterial T7 polymerase) with lipofectamine 2000 (Invitrogen). All plasmids were transfected at a concentration of 10 ng/mL. Twenty-four hours after transfection, the media was replaced either by conditional media from normal L cells (control, ATCC: CRL-2648) or from a WNT3A producing L cell line (ATCC: CRL-2647). Firefly and Renilla luciferase activities were measured 24 h later using the Dual-Glo luciferase reporter assay system (Promega) with an Ascent Lunimoskan luminometer (Labsystems). Firefly luciferase activity was normalized to the constitutive Renilla luciferase activity.

Protein production and purification

Conditioned media was harvested from transfected cultures, pooled and filtered through 0.2 µm filters and stored at 4˚C until use.

Prey protein transfections were quantified by β-lactamase assay, measuring the turnover of nitrocefin substrate by changing absorbance at 485 nm over time *(63)*, then normalized by dilution.

Biotinylated bait ectodomain transfections were dialyzed against PBS using SnakeSkin dialysis tubing (molecular weight cut-off 10,000 Da; Thermo Scientific) and several buffer changes (approximately 25 – 30 L in total). Biotinylated protein concentration was quantified by ELISA, using streptavidin-coated microplates and a monoclonal antibody to detect the CD4d3+4 tag *(63)* (Nunc Immobilizer, Thermo Fisher Scientific).

Unbiotinylated ectodomain transfections were collected and quantified by ELISA using nickel-coated microplates and pooled for purification using nickel-sepharose columns (HisTrap HP, GE Healthcare, Chicago, IL, USA) and an AKTAxpress chromatography system (GE Healthcare). Briefly, nickel-charged columns were pre-eluted with elution buffer (10 mM Na_2HPO_4, 10 mM NaH_2PO_4, 0.5 M NaCl, 0.4 M imidazole, pH 7.4, filtered and degassed under vacuum) then equilibrated with running buffer (10 mM Na_2HPO_4, 10 mM NaH_2PO_4, 0.5 M NaCl, 0.04 M imidazole, pH 7.4, filtered and degassed under vacuum). Pooled supernatants were adjusted to approximately 0.1 M NaCl and 0.01 M imidazole then run through the column at a flow rate of 1 mL/min. The column was washed with 15 volumes of running buffer and then eluted in 0.5 mL fractions with 10 columns of elution buffer. Peak

This is the author's version of the work. It is posted here by permission of the AAAS for personal use, not for redistribution. The definitive version was published in *Science* on 03/05/2024, DOI: 10.1126/science.ade6970.

fractions were pooled and dialyzed against PBS, then quantified by absorption at 280 nm using a Nanodrop 1000 instrument (Thermo Fisher Scientific).

Proteins used in surface plasmon resonance and crystallography were purified from conditioned medium collected from stable cell line cultures. The media was buffer exchanged with PBS and His-tagged proteins were captured with 5 mL HisTrap Excel columns (GE Healthcare), washed with 20 mM imidazole and eluted with 300 mM imidazole containing PBS buffer. The eluted proteins were further purified using a Superdex 200 16/60 column (GE Healthcare), in a buffer of 10 mM HEPES, pH 7.4, 150 mM NaCl. Before crystallization, purified glycoproteins were deglycosylated using EndoF1.

Antibody generation and purification

To characterize the expression pattern of the receptor protein, we raised and affinity purified a polyclonal antibody against the recombinant extracellular domain of zebrafish Cachd1.

Briefly, purified zebrafish Cachd1 ectodomain was prepared (see above) and sent to Cambridge Research Biochemicals (Billingham, United Kingdom) for a rabbit immunization protocol. Activity against the Cachd1 ectodomain in rabbit blood sera was confirmed by ELISA. The blood serum was then affinity purified against biotinylated recombinant ectodomain immobilized on a streptavidin sepharose column, using an AKTAxpress chromatography system. Purified antibodies were eluted in fractions using a low pH buffer, then immediately neutralized. Peak fractions were tested for anti-Cachd1 activity, then pooled and dialyzed against PBS. Total protein concentration was determined by absorbance at 280 nm by Nanodrop. The affinity purified antibody was checked for purity by SDS-PAGE and then validated by western blot, immunohistochemistry and flow cytometry (see Fig. S1) *(38)*.

Retrogenix Cell Microarray Technology

Cell Microarray Technology *(71)* was used to identify potential binding partners for multimerized human CACHD1 ectodomain (prepared as above) and was performed by Charles River Discovery Research Services UK Limited (formerly Retrogenix Limited; Chinley, United Kingdom; for bait target details, see *(72)*).

Flow cytometry

To test Cachd1 prey binding interactions, live suspension culture HEK293E cells transfected with *FZD-EGFP* constructs (and mock transfected control cells) were split into samples of 2.5-5.0 × 10^5 cells in 1% BSA in PBS and placed in individual wells of 96 well round bottomed culture plates on ice. The cells were collected by centrifugation (200 × g for 5 minutes at 4°C) and then resuspended in dilutions of prey protein (human or zebrafish Cachd1 and mouse CD200R; batchwise dilution determined by β-lactamase assay, see above) or 1% BSA in PBS (secondary only controls), and incubated on ice for 30 minutes. The cells were washed three times, by centrifugation and resuspension in PBS, then incubated in anti-FLAG-phycoerythrin secondary antibody diluted in 1% BSA in PBS (Antibody registry ID: AB_1268475, mouse IgG1, 1:500, Abcam) for a further 30 minutes. Cells were washed three times, by centrifugation and resuspension in PBS, then analyzed using a LSRFortessa flow cytometer with a 5-decade logarithmic scale for detection, a high throughput sampler for 96 well plates and FACSDiva software (BD Biosciences).

Where possible, we verified the surface expression of bait proteins or EGFP-tagged Cachd1 in live cultures by following the same protocol but using specific primary antibodies in place

This is the author's version of the work. It is posted here by permission of the AAAS for personal use, not for redistribution. The definitive version was published in *Science* on 03/05/2024, DOI: 10.1126/science.ade6970.

of prey proteins (Fig. S1A, S14E; anti-Cachd1, diluted 1:700, bespoke rabbit polyclonal, see above; OMP-18R5, human anti-FZD7 IgG *(33)*, diluted 1:2000, OncoMed Pharmaceuticals; anti-Smo, AB_1270802, rabbit polyclonal, diluted 1:500, Abcam; anti-Jamb, bespoke goat polyclonal, diluted 1:200, Everest Biotech) and Alexa Fluor-conjugated secondary antibodies (Molecular Probes, diluted 1:500).

The same procedure was followed for experiments testing the ability of OMP-18R5 to block Cachd1 prey-FZD-EGFP interactions, but with an additional incubation step before the application of prey proteins: cells were resuspended in OMP-18R5 diluted in 1% BSA in PBS (1:800) or 1% BSA in PBS only (control) and incubated for 30 minutes on ice, washed three times in PBS, and then resuspended in prey protein dilutions.

Mock transfection controls were used to determine forward and side scatter voltages for samples prior to data collection, and for background gating thresholds in data analysis. "Cells only" (no prey/primary or secondary antibodies) and "secondary antibody only" controls were included in every experiment. Flow cytometry data was analyzed using FlowJo V10 (FlowJo, Ashland, OR, USA). Single cell populations were isolated using forward and side scatter values, bisected into EGFP-negative (untransfected) and EGFP-positive subpopulations and then the median value for phycoerythrin fluorescence (indicating prey binding) calculated for each (Fig. S14B). Binding of prey protein to EGFP-positive cells was quantified by taking the ratio of the medians: $\Delta M_{PE} = \ln\left(M_{PE}^{EGFP+}/M_{PE}^{EGFP-}\right)$.

Surface Plasmon Resonance

Biotinylated proteins ($FZD5/7/8_{CRD}$ and $LRP6_{P1E1P2E2}$ or $LRP6_{P3E3P4E4}$) were obtained by co-transfection of avi3-tagged constructs *(73)* and a BirA-ER plasmid into HEK293T cells. About 500-1,000 resonance units of each of the biotinylated proteins were immobilized on a SA sensor chip (GE Healthcare), using a Biacore S200 machine (GE Healthcare) at 25 °C with a running buffer comprising 10 mM HEPES, pH 7.5, 150 mM NaCl and 0.005% Tween 20. A dilution series of purified $CACHD1_{ECD}$ analyte was passed over the flow cells at high flow rate (100 µL/min) and the real-time response recorded at a frequency of 10 Hz. The response was plotted versus the concentration of the analyte and fitted by nonlinear regression to a one-site saturation binding model (Sigma Plot, Systat software, Inc. San Jose, CA).

Crystallization, data collection and structure determination

$CACHD1_{ECD}$ was concentrated to 5 mg/mL and mixed with equal molar of $FZD5_{CRD}$ and $LRP6_{P3E3P4E4}$. Crystallization screening was carried out using the sitting-drop vapor diffusion method in 96-well plates. The crystals were obtained in conditions of 0.1 M Calcium acetate; 0.1 M Sodium acetate, pH 4.5; 10% (w/v) PEG 4000.

Crystals were flash frozen by immersion in a reservoir solution supplemented with 25% (v/v) glycerol followed by transfer to liquid nitrogen, and kept at −173 °C during X-ray data collection at I03, Diamond Light Source, with a wavelength of 0.9762 Å. The best diffracted crystal shows resolution of 4.7 Å, with space groups of $C2_1$. Structure determination by molecular replacement with components structures solved in our laboratory and refinement used PHENIX *(74)* to good R factors and bond angles (see Table S2 for data collection and refinement statistics). The ternary complex structure was deposited at the Research Collaboratory for Structural Bioinformatics Protein Data Bank under accession number 8S7C.

This is the author's version of the work. It is posted here by permission of the AAAS for personal use, not for redistribution. The definitive version was published in *Science* on 03/05/2024, DOI: 10.1126/science.ade6970.

In situ hybridization and Immunohistochemistry

Embryos or larvae were fixed in 4% paraformaldehyde and *in situs* performed following standard protocols *(75)*. To create plasmid templates for *in situ* probe generation, regions of the *zgc:101731*, *slc18a3b*, *aoc1* and *cachd1* genes were PCR-amplified (see Table S5 for primer sequences) and TA-cloned into the *pCRII* vector. The *kiss1 in situ* probe template was generated directly by PCR (see Table S5). Previously published *in situ* probes used include (see Table S7 for references): *kctd12.1*, *kctd12.2*, *kctd8*, *axin2*, *lft1*, *otx5*, *spaw*, *selenop2*, *prss1*, *aldh1a3*, *dbx1b*, *wnt3a*, *lef1*. All enzymes used for plasmid linearization and *in vitro* transcription are listed in Table S7. Antisense probes were generated with digoxigenin and fluorescein labelling kits (Roche). Anti-digoxigenin-AP and anti-fluorescein-AP antibodies (Antibody registry ID: AB_2734716 and AB_2734723 respectively, Roche) coupled with 5-bromo-4-chloro-3′-indolyphosphate and nitro-blue tetrazolium chloride were used to visualize colorimetric *in situs*. Anti-digoxigenin-POD and anti-fluorescein-POD antibodies (AB_514500 and AB_840257 respectively, Roche) and Alexa Fluor-conjugated tyramides (Molecular Probes) were utilized for detection in fluorescent *in situ* hybridization.

In situ hybridization chain reaction was performed according to published protocol *(76)* using Alexa Fluor-conjugated hairpin amplifiers and hybridization buffers from Molecular Instruments Inc. (Los Angeles, CA, USA). Probe sets for *cachd1* and *lrp6* are detailed in Table S8.

For immunohistochemistry, embryos were stained according to published protocol, except for using freshly fixed embryos without storage in methanol *(77)*. Antibodies used in this study were: anti-acetylated α-tubulin (Antibody registry ID: AB_477585, clone 6-11B-1, mouse IgG2b, Sigma, diluted 1:250 in blocking solution), anti-SV2 (AB_2315387, mouse IgG1, deposited to the Developmental Studies Hybridoma Bank by Buckley, K.M., diluted 1:250), anti-HuC/HuD (AB_221448, clone 16A11, mouse IgG2b, Molecular Probes, diluted 1:250), anti-phospho-S10-histone H3 (AB_443110, mouse IgG1, Abcam, diluted 1:250), anti-Cachd1 (this study, rabbit polyclonal, diluted 1:50), anti-GFP (AB_10013661, rabbit polyclonal, Torrey Pines Biolab, diluted 1:1000; or AB_300798, chicken polyclonal, Abcam, diluted 1:500). The use of anti-PCNA (AB_2160343, clone PC10, mouse IgG2a, Cell Signaling Technology, diluted 1:100) required heat-mediated antigen retrieval: embryos were incubated in 10 mM Sodium citrate in PBS, pH 6.0, at 85°C for 20 mins before blocking. Alexa Fluor-conjugated anti-mouse IgG subtypes/rabbit/chicken secondary antibodies (Molecular Probes) were diluted 1:200 in blocking solution before use. 4′,6-diamidino-2-phenylindole (DAPI, Invitrogen) was added to embryos (10 μg/mL in PBST) to counterstain nuclei before imaging.

Quantitative RT-PCR

RNA was extracted from cell culture or organoids according to the manufacturer’s instructions (Qiagen RNeasy; Qiagen). cDNA was prepared using Maxima first strand cDNA synthesis kit with dsDNase (#1672, Thermo Fisher Scientific). Quantitative PCR detection was performed using PowerUp SYBR Green Master Mix (A25742, Applied Biosystems, Waltham, MA, USA). Assays for each sample were done in triplicate and were normalized to housekeeping genes *ACTB* (human *β-ACTIN*) or *Hrpt1* (mouse). Primer sequences are listed in Table S5.

Heat shock, laser cell ablation, BrdU, labelling of habenular projections and transplantation experiments

This is the author's version of the work. It is posted here by permission of the AAAS for personal use, not for redistribution. The definitive version was published in *Science* on 03/05/2024, DOI: 10.1126/science.ade6970.

For rescue experiments, embryos transgenic for *Tg(HSE:cachd1, EGFP)w160* were heat shocked for 30 minutes in a 40˚C water bath, then raised at standard temperature to 4 dpf and fixed in 4% paraformaldehyde.

Laser cell ablation, BrdU incorporation experiments and lipophilic dye (DiI/DiD) labelling of habenular efferent projections were performed as previously described *(19)*.

Transplantation experiments were also done as previously described *(19)* using embryos from *Et(gata2a:EGFP)pku588*, *cachd1*$^{u761/+}$ incrosses as donors and fixing host embryos at 56 hpf or 4 dpf.

Imaging

For transmitted light pictures, larvae were mounted in glycerol and imaged using differential interference contrast optics (Leica CTR6000; 20× and 40× objectives; Leica Microsystems, Wetzlar, Germany). For confocal microscopy, heads were mounted in 1.2% low-melt agarose in glass-bottom dishes (MatTek, Ashland, MA, USA or LabTek, Grand Rapids, MI USA). Fluorescence was imaged by confocal laser scanning microscopy (Leica TCS SP5 and Leica TCS SP8) using a 40× oil-immersion objective (40× 1.3 Oil DIC III) or a 25× water-immersion objective (25× 0.95), and z stacks were acquired in 0.75 – 2 µm intervals. Cell cultures were imaged using a Marianis Spinning Disk (Intelligent Imaging Innovations, Inc., Denver, CO USA) system. 3D reconstructions and maximum-intensity projections were generated from stacks of images with Volocity (Improvision, Coventry, UK) and ImageJ (NIH). Image segmentation and quantification was performed using IMARIS (v8.0.1, Bitplane, Zurich, Switzerland).

Statistics

Statistical analysis was performed using RStudio (v1.4.1106, base R x64 v4.0.5, DescTools package v0.99.44) and Microsoft Excel 2010. Charts were plotted using the ggplot2 package (v3.3.3).

Descriptive statistics, scatterplots and normalized histograms for flow cytometry experiments were generated in FlowJo V10 (FlowJo, Ashland, OR, USA).

The Q′ test for equal proportions and modified Marascuilo procedure for multiple testing (using a Wilson variance calculation) are described in *(78)*. Where the proportion was $0.1 < \hat{p} < 0.9$ and/or $n > 20$, confidence intervals were calculated using a normal assumption; otherwise by the Wilson count method. Fisher’s exact tests were used in place of χ^2 where expected values were below 5.

Manuscript and figure preparation

The manuscript was prepared according to the ARRIVE guidelines for reporting animal research *(79)*.

Figures were compiled using Adobe Photoshop CS6 (64 bit).

Fluorescent and confocal microscopy images were adjusted globally for brightness and contrast using FIJI (v1.53n), scale bars added and then flattened into RGB images and exported as TIFFs.

Color balance of wholemount *in situ* hybridization images was adjusted in Adobe Photoshop CS6 (64 bit).

This is the author's version of the work. It is posted here by permission of the AAAS for personal use, not for redistribution. The definitive version was published in *Science* on 03/05/2024, DOI: 10.1126/science.ade6970.

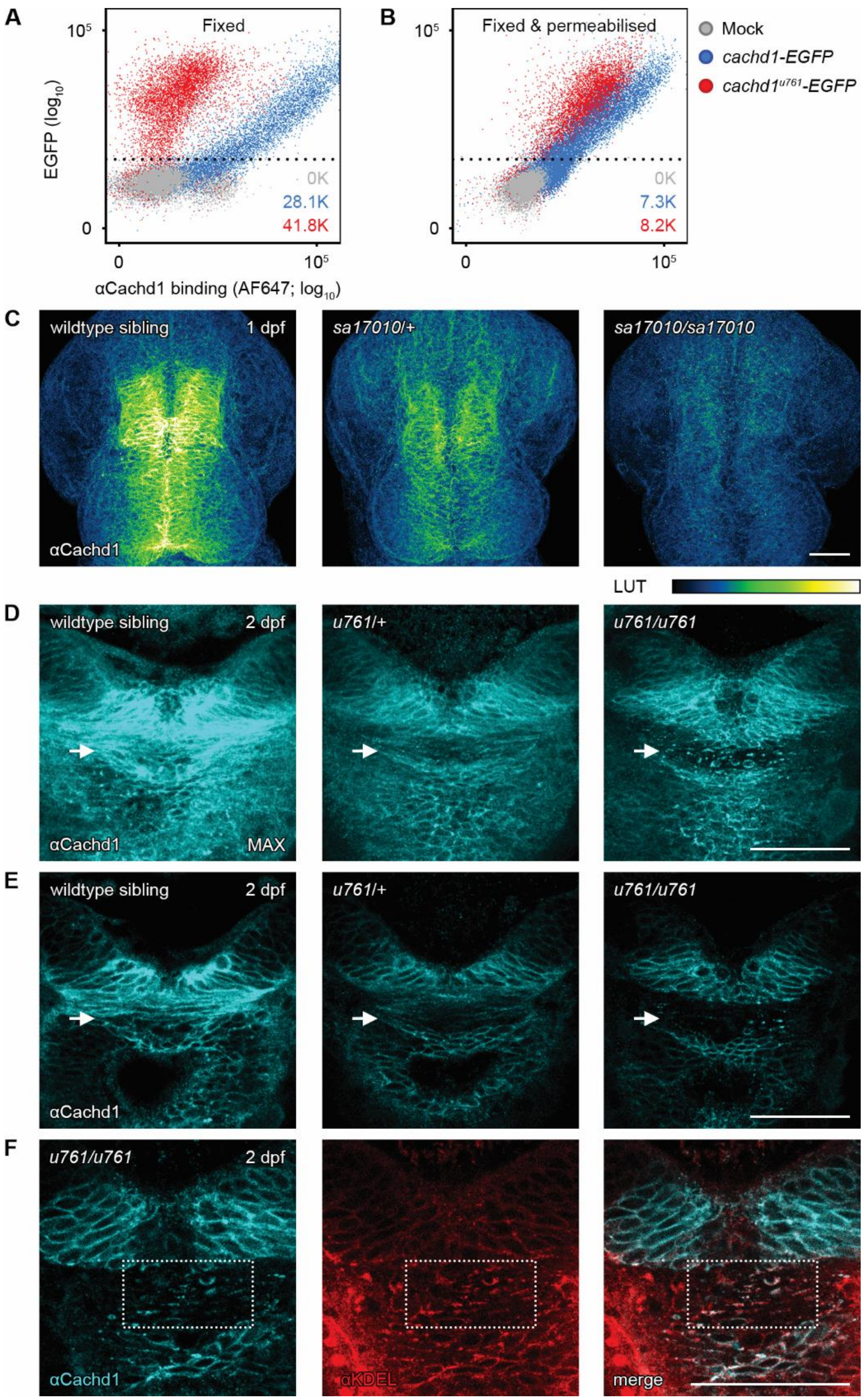

Fig. S1. Cross-validation of *cachd1* mutations and anti-Cachd1 antibody.
(**A** and **B**) Flow cytometry of untransfected (Mock, grey) and transfected HEK293E cells expressing wildtype Cachd1- (blue) or mutant Cachd1^{u761}-EGFP (red) fusion proteins stained with anti-Cachd1 antibody after fixation (A) and permeabilization (B). The mutant fusion protein was stained strongly after permeabilization of the cells, suggesting the protein was not trafficked to the plasma membrane. Numbers in each panel indicate the total number of EGFP-positive events recorded. (**C**) Dorsal views of brains of 1 dpf siblings from a *cachd1*$^{sa17010/+}$ incross,

This is the author's version of the work. It is posted here by permission of the AAAS for personal use, not for redistribution. The definitive version was published in *Science* on 03/05/2024, DOI: 10.1126/science.ade6970.

stained with anti-Cachd1 antibody. Note that the *sa17010* allele has an early nonsense mutation that is expected to prevent translation of the protein. Intensity of staining of the midbrain roofplate and dorsal diencephalon is correlated to *sa17010* genotype (see Table S1), indicating the antibody is specific for Cachd1. Maximum projections of confocal stacks. (**D**, **E** and **F**) Dorsal views of 2 dpf *cachd1*$^{u761/+}$ incross embryos stained with anti-Cachd1 antibody (D, E and F; cyan) and anti-KDEL (F; red). Note the punctate expression in the posterior commissure (white arrows in D, D′) in *u761* homozygotes, and the colocalization with ER resident proteins, marked by the anti-KDEL antibody (dotted box in F), indicating retention of the mutant protein. Representative maximum projection (D), single plane images from the same confocal stacks (E) or single plane confocal images (F). Scale bars = 50 µm.

This is the author's version of the work. It is posted here by permission of the AAAS for personal use, not for redistribution. The definitive version was published in *Science* on 03/05/2024, DOI: 10.1126/science.ade6970.

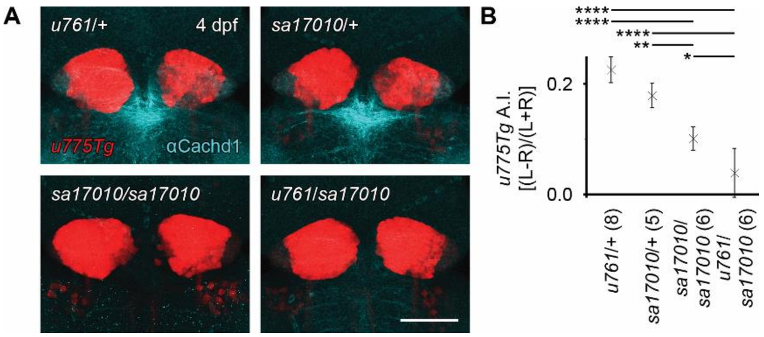

Fig. S2. The *sa17010* allele of *cachd1* phenocopies, and is unable to complement, *u761*.
(**A**) Dorsal views of 4 dpf *Tg(gng8:EGFP)u775/o* larvae carrying *u761* and/or *sa17010* alleles of *cachd1*, stained with anti-Cachd1 antibody (cyan). *Tg(gng8:EGFP)u775* expresses EGFP in differentiated dorsal habenula neurons (red). Right habenula labelling is larger in the *sa17010* and *u761/sa17010* larvae than in heterozygotes, indicating bilateral 'double left' symmetry. Maximum projections of confocal stacks. Scale bar = 50 µm. (**B**) Asymmetry index calculated from EGFP habenulae volumes of *u761/+*, *sa17010/+*, *sa17010/sa17010* and *u761/sa17010* 4 dpf larvae. *sa17010* homozygotes and *u761/sa17010* transheterozygotes are bilaterally symmetric, suggesting loss of function of *cachd1* is causative of the *rorschach* phenotype. Number of larvae analyzed indicated in brackets. Error bars represent 95% confidence intervals of the mean. ANOVA (degrees of freedom = 3, $F = 27.41$, $P = 1.9 \times 10^{-7}$) and *post hoc* Tukey pairwise comparisons was used for hypothesis testing, * $0.1 \geq P > 0.05$, ** $0.05 \geq P > 0.01$, **** $P \leq 0.005$.

This is the author's version of the work. It is posted here by permission of the AAAS for personal use, not for redistribution. The definitive version was published in *Science* on 03/05/2024, DOI: 10.1126/science.ade6970.

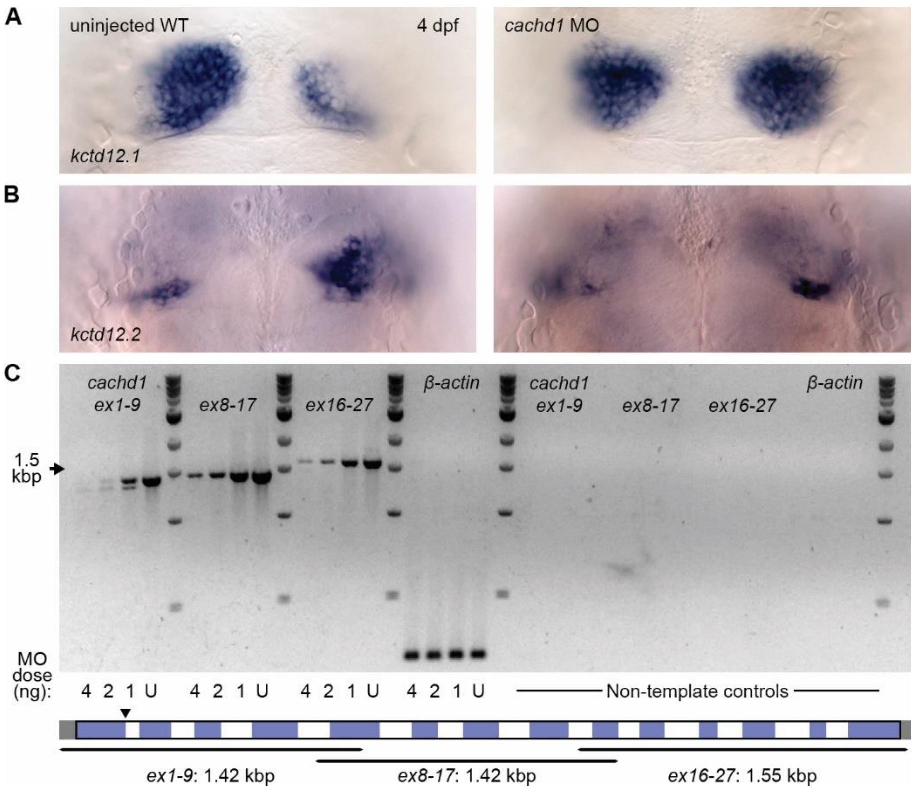

Fig. S3. Morpholino knockdown of *cachd1* results in bilateral symmetry.
(**A**-**B**) Dorsal views of 4 dpf uninjected wildtype and *cachd1* morpholino-injected larvae after wholemount *in situ* hybridization using antisense riboprobes against asymmetric dorsal habenula markers *kctd12.1* (A, n = 253/263) or *kctd12.2* (B, n = 16/17). Note the increase in *kctd12.1* expression and corresponding decrease of *kctd12.2* expression in the right habenula of *cachd1* morphants. (**C**) Semi-quantitative RT-PCR for *cachd1* transcripts (three primer sets spanning exons 1-9, exons 8-17 and exons 16-27) and reference gene *β–actin* in uninjected embryos (U) and those injected with ~4 ng, 2 ng and 1 ng of *cachd1* morpholino (MO1) showing a dose dependent reduction in *cachd1* expression and mis-splicing (exon 1-9). Subsequent Sanger sequencing of the RT-PCR products indicated mis-splicing resulted in an 89 bp deletion from the 3’ end of exon 1 and usage of a cryptic donor site. Bottom panel: Schematic of *cachd1* transcript structure (ENSDART00000087964 Zv11, approximately to scale; alternating exons in blue/white and grey unannotated UTRs) with expected sizes of RT-PCR products indicated. The splice junction targeted by MO1 is indicated with a black arrowhead.

This is the author's version of the work. It is posted here by permission of the AAAS for personal use, not for redistribution. The definitive version was published in *Science* on 03/05/2024, DOI: 10.1126/science.ade6970.

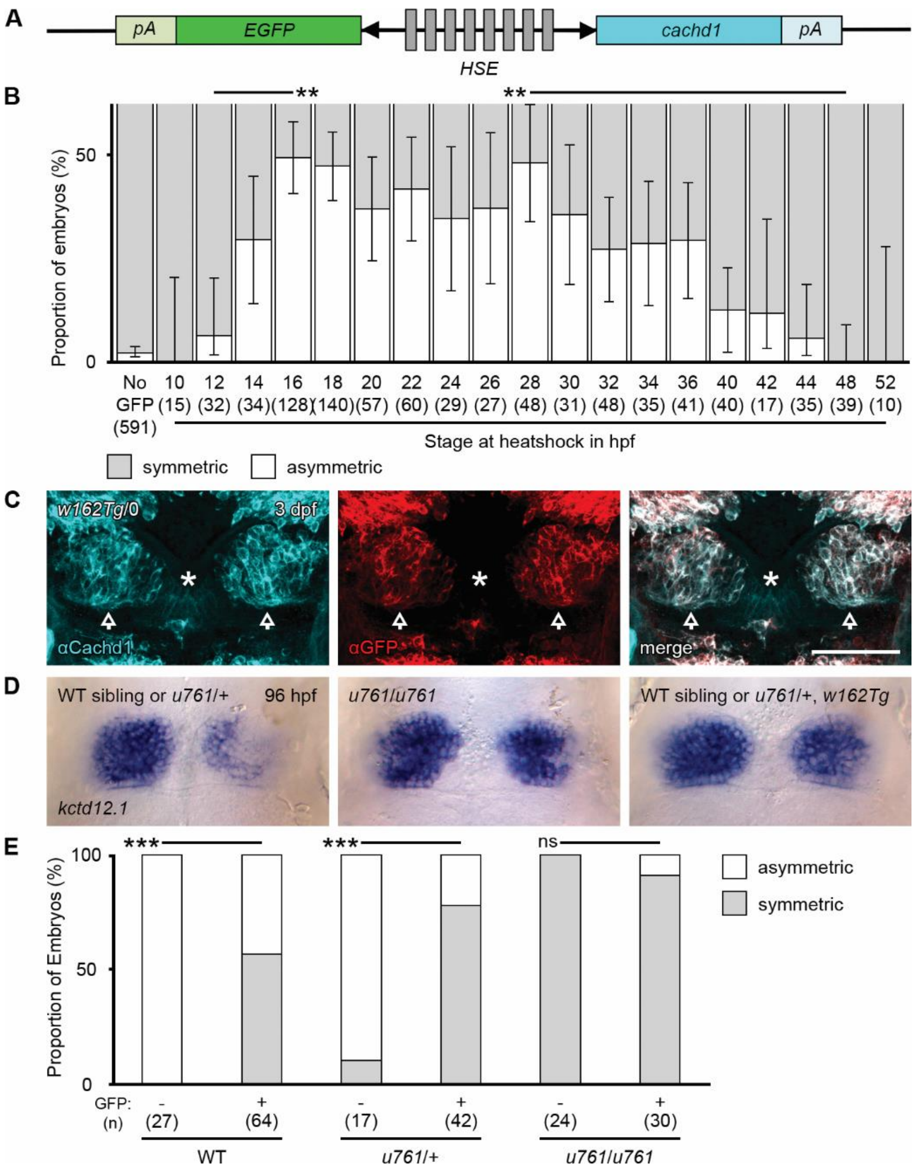

Fig. S4. Asymmetry in *cachd1*^{u761} mutants is restored by global overexpression of wildtype *cachd1*, but not in post-mitotic neurons.

(**A**) Schematic of construct used to generate transgenic *Tg(HSE:cachd1, EGFP)w160* fish. A bidirectional heat shock promoter (HSE) drives simultaneous expression of EGFP and Cachd1 in response to acute exposure to heat. (**B**) Chart showing the percentage of GFP-positive, *cachd1*^{u761} mutant larvae with symmetric (grey) or asymmetric (white) *kctd12.1* expression at 4 dpf, after receiving a heat shock at the stage indicated (GFP-negative, heat-shocked sibling larvae included in 'No GFP' column). There was substantial restoration of asymmetry after heat shock between 16 - 28 hpf, after which the proportion of larvae with wildtype phenotype declined. Number of larvae indicated in brackets. Error bars represent 95% confidence interval for the proportion, calculated using a normal approximation, or Wilson score when the proportion was less than 0.1, and/or the number of larvae tested was less than 20. Q′ test of equality of proportions (degrees of freedom = 19, $\chi^2 = 342.27$, $P = 4.0 \times 10^{-61}$) and *post hoc* modified Marascuilo procedure for multiple comparisons of proportions with Benjamini-Hochberg correction for multiple testing was used to test significance, ** $0.05 \geq P > 0.01$. A

This is the author's version of the work. It is posted here by permission of the AAAS for personal use, not for redistribution. The definitive version was published in *Science* on 03/05/2024, DOI: 10.1126/science.ade6970.

limited number of statistically significant differences are presented here for clarity. (**C**) Dorsal views of *Tg(neurod1:cachd1-EGFP)w162* 3 dpf larvae stained with antibodies against Cachd1 and GFP. Ectopic expression of EGFP-tagged Cachd1 protein in post-mitotic neurons is driven by a *neurod1* promoter. Note the endogenous Cachd1 protein expression in the periventricular zone (asterisk) that does not co-localize with GFP antibody labelling in the dorsal habenulae (arrowheads). Representative maximum projections of confocal stacks. Scale bar = 50 µm. (**D**) Representative images of *in situ* hybridization with *kctd12.1* riboprobe in 96 hpf wildtype or *u761* heterozygous siblings (left panel), *u761* mutants (middle panel) or *w162Tg* transgenic, wildtype or *u761* heterozygotes (right panel). Note that overexpression of exogenous Cachd1-EGFP did not rescue the *u761* symmetry phenotype, and also resulted in bilateral symmetry in wildtype and heterozygous siblings. (**E**) Quantification of *kctd12.1 in situ* asymmetry phenotype in embryos expressing (+) or not expressing (-) the *neurod1:cachd1-EGFP* transgene. Number of embryos for each condition is brackets below each bar. Fisher's exact test, *** $0.01 \geq P > 0.005$.

This is the author's version of the work. It is posted here by permission of the AAAS for personal use, not for redistribution. The definitive version was published in *Science* on 03/05/2024, DOI: 10.1126/science.ade6970.

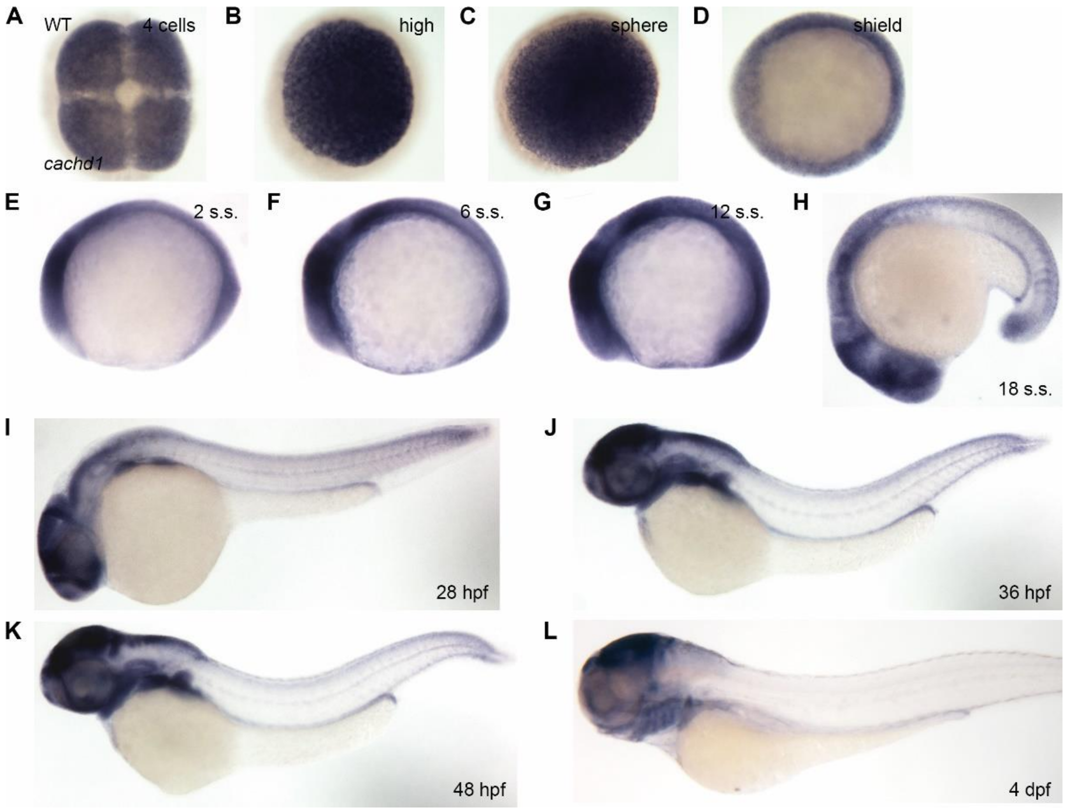

Fig. S5. Spatiotemporal pattern of *cachd1* gene expression.
(**A-L**) Representative images of colorimetric *in situ* hybridization with *cachd1* antisense riboprobe using whole wildtype embryos at different stages (indicated in each panel). Abbreviation s.s.: somite stage.

This is the author's version of the work. It is posted here by permission of the AAAS for personal use, not for redistribution. The definitive version was published in *Science* on 03/05/2024, DOI: 10.1126/science.ade6970.

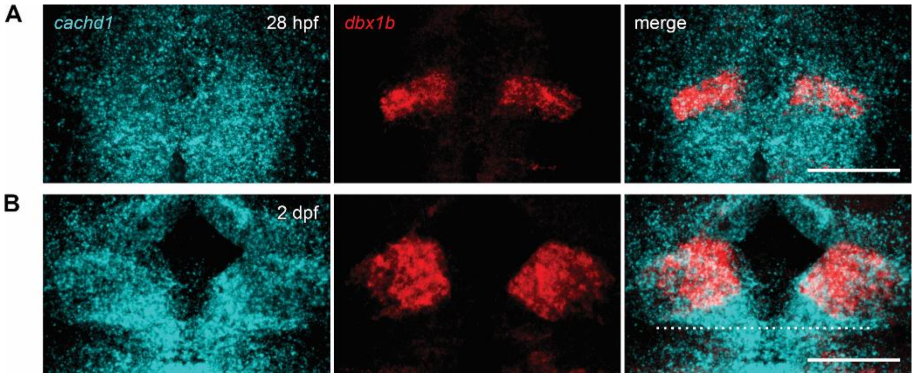

Fig. S6. *cachd1* is expressed by presumptive habenula neuron progenitors.
(**A**, **B**) Dorsal views of habenulae after double fluorescent *in situ* hybridization with *cachd1* antisense riboprobe (cyan) and the habenula neuron progenitor marker *dbx1b* (red) at 28 hpf (A) and 2 dpf (B). A single dotted line in (B) indicates the approximate position of the posterior commissure. Maximum projections of confocal stacks. Scale bars = 50 µm.

This is the author's version of the work. It is posted here by permission of the AAAS for personal use, not for redistribution. The definitive version was published in *Science* on 03/05/2024, DOI: 10.1126/science.ade6970.

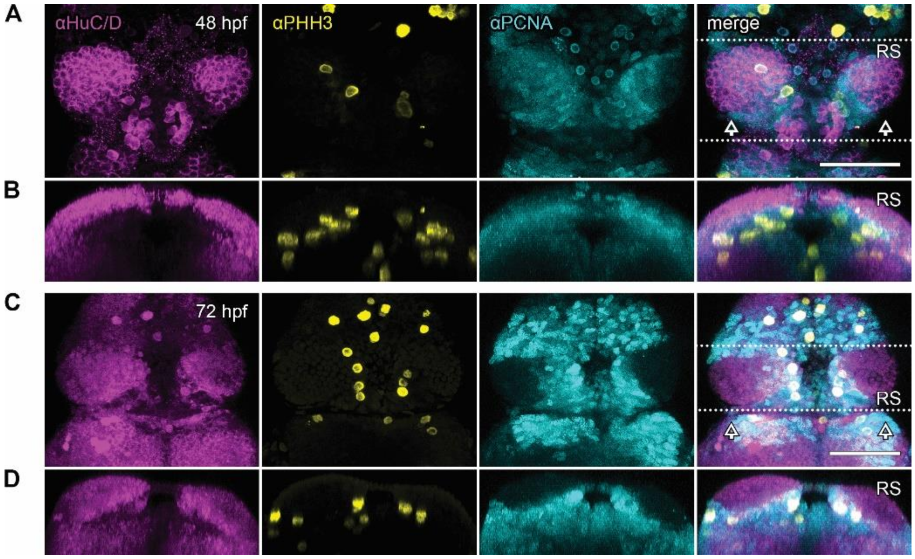

Fig. S7. The periventricular domain ventral to the pineal complex is proliferative.
(**A**-**D**) Dorsal (A, C) and transverse projections (B, D) of 48 hpf (A, B) and 72 hpf (C, D) embryos stained with anti-HuC/D to mark differentiated neurons (magenta), anti-phospho-histone H3 to mark neuronal cells in M-phase (yellow) and anti-PCNA to mark neuronal cells in G1/S phase (cyan) of the cell cycle. Maximum projections of confocal stacks. The positions of habenulae are indicated with open arrowheads. The dotted lines in (A, C) indicate the volume shown in the transverse projections (B, D). Note that staining with anti-PCNA required antigen retrieval steps that inhibited anti-Cachd1 labelling, preventing co-staining; compare to Cachd1 expression in Fig. 1, S1, S2, S6. Scale bars = 50 µm.

This is the author's version of the work. It is posted here by permission of the AAAS for personal use, not for redistribution. The definitive version was published in *Science* on 03/05/2024, DOI: 10.1126/science.ade6970.

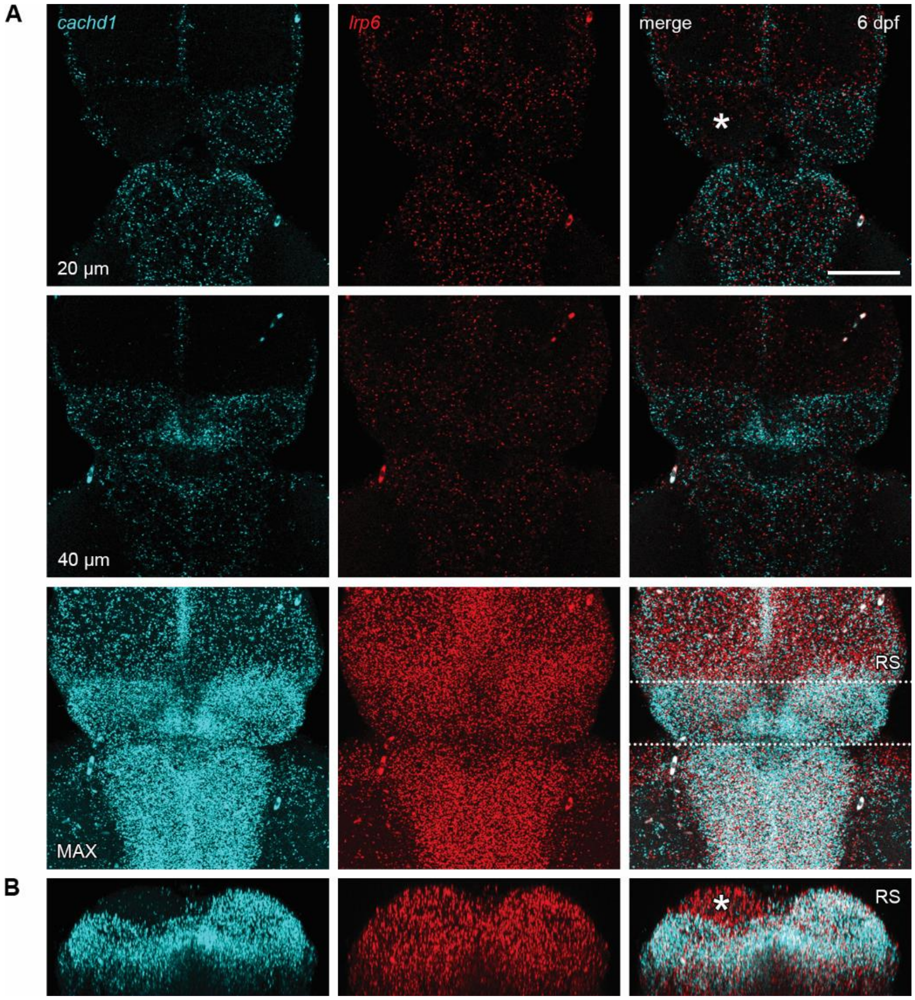

Fig. S8. Expression of *cachd1* is asymmetric at larval stages.
(**A-B**) Dorsal view (A) and transverse projection (B) of a dissected 6 dpf wildtype larva after *in situ* hybridization chain reaction with probes against *cachd1* (cyan) and *lrp6* (red).
(A) Single confocal slices at ~20 µm and 40 µm depth (from dorsal brain surface) and a maximum projection of the same confocal stack. Dotted lines indicate the approximate volume presented in the transverse projection (RS, B). *cachd1* expression remains in the periventricular habenular domain ventral to the pineal but is also expressed in the right habenula in its entirety; it is absent from the dorsal-most domain of the left dorsal habenula (asterisk). *lrp6* is expressed ubiquitously. Scale bar = 50 µm.

This is the author's version of the work. It is posted here by permission of the AAAS for personal use, not for redistribution. The definitive version was published in *Science* on 03/05/2024, DOI: 10.1126/science.ade6970.

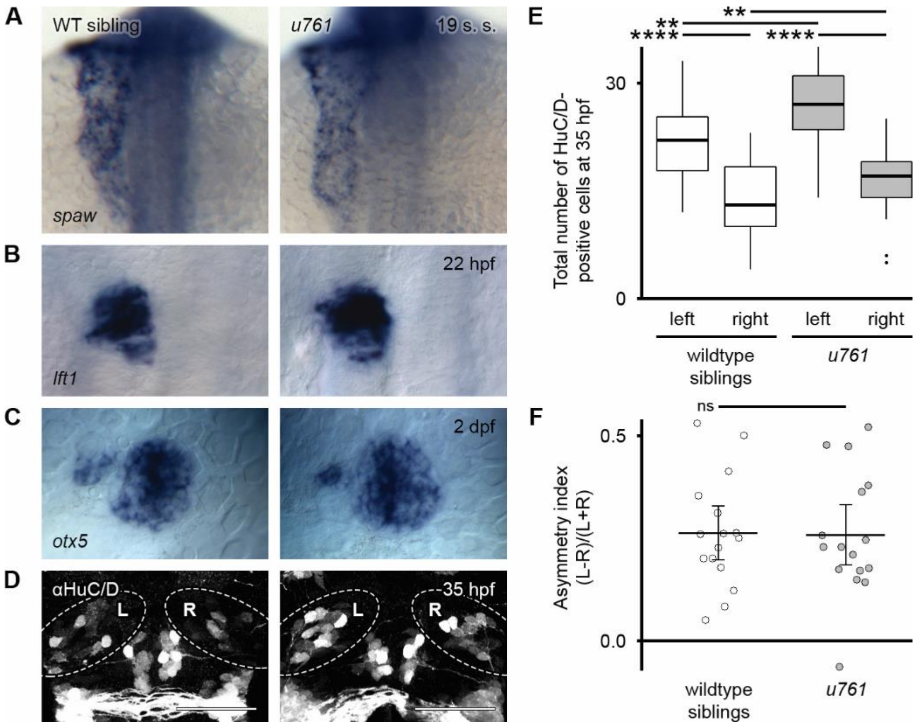

Fig. S9. Early Nodal-related left-right asymmetries are unperturbed in *cachd1^{u761}* mutants. (**A**-**C**) Dorsal views of wildtype sibling and *cachd1^{u761}* mutant embryos after colorimetric wholemount *in situ* hybridization with antisense riboprobes for the *nodal* signaling pathway ligand-encoding gene *spaw* (A, 19 somite stage) or target, *lft1* (B, 22 hpf), or pan-pineal complex marker *otx5* (C, 2 dpf) indicating early asymmetric expression of these marker genes is unaffected in *cachd1^{u761}* mutants. (**D**) Dorsal views of wildtype or *cachd1^{u761}* mutant embryos stained with anti-HuC/D to mark differentiated neurons at 35 hpf. Left and right dorsal habenula indicated with dotted lines. Note the overall increase in the number of differentiated neurons in the left and right dorsal habenula of *cachd1^{u761}* mutants (quantified in E). Maximum projections of confocal stacks. Scale bars = 50 µm. (**E**) Boxplots showing quantification of the number of anti-HuC/D-positive nuclei in the left and right dorsal diencephalon of wildtype siblings (white) and *cachd1^{u761}* mutants (grey); n = 16 for both groups. Kruskal-Wallis rank sum test (degrees of freedom = 3, $\chi^2 = 27.21$, $P = 5.3 \times 10^{-6}$) and *post hoc* pairwise comparisons using Wilcoxon rank sum test with continuity correction and Benjamini-Hochberg correction for multiple testing, ** $0.05 \geq P > 0.01$, **** $P \leq 0.005$. (**F**) Dot plots of the asymmetry index calculated for each wildtype sibling and *cachd1^{u761}* mutant embryo, based on the number of anti-HuC/D-positive nuclei. Although there is an overall increase in early neurogenesis in *cachd1^{u761}* mutants, there is a leftward bias, consistent with correct early Nodal-related asymmetry determination. Bar indicates sample mean and error bars indicate 95% confidence intervals of the mean. Welch two sample, two-tailed, *t*-test (degrees of freedom = 29.629, $t = 0.094$, $P = 0.926$), ‘ns’ $P > 0.1$.

This is the author's version of the work. It is posted here by permission of the AAAS for personal use, not for redistribution. The definitive version was published in *Science* on 03/05/2024, DOI: 10.1126/science.ade6970.

A

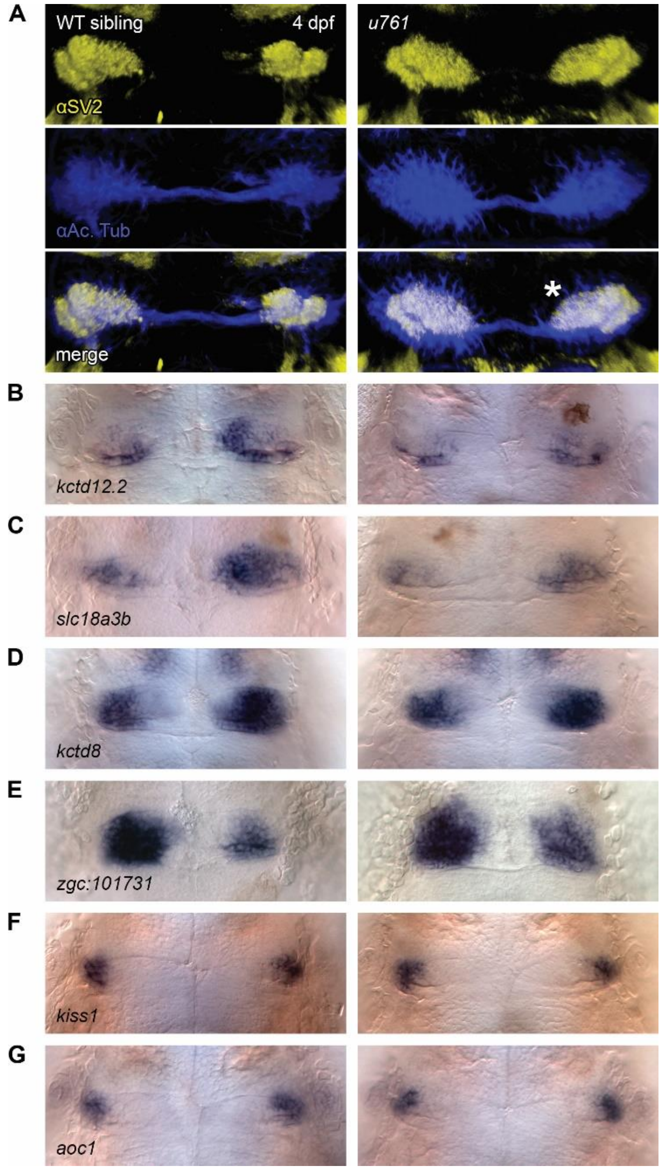

Fig. S10. Symmetric neuroanatomy and gene expression in the dorsal habenulae of *cachd1*^{u761} mutants.

(**A**) Dorsal views of immunohistochemistry labelling neuropil (anti-SV2, yellow) and axons (anti-acetylated tubulin, blue) in the habenulae in 4 dpf wildtype and *cachd1*^{u761} mutant larvae. Asterisk marks the increased volume of dHb_L-associated neuropil in the right habenula of *cachd1*^{u761} mutants. Maximum projections of confocal stacks. (**B-G**) Dorsal views of 4 dpf wildtype and *cachd1*^{u761} larvae after colorimetric wholemount *in situ* hybridization with riboprobes against dorsal habenula markers *kctd12.2* (B), *slc18a3b* (C), *kctd8* (D), *zgc*:101731 (E) and ventral habenula markers *kiss1* (F) and *aoc1* (G). The asymmetric dorsal habenula markers are reduced or symmetric in *cachd1*^{u761} mutants, but the ventral habenula markers are unaffected, suggesting *cachd1* does not play a role in neurogenesis of the ventral habenula.

This is the author's version of the work. It is posted here by permission of the AAAS for personal use, not for redistribution. The definitive version was published in *Science* on 03/05/2024, DOI: 10.1126/science.ade6970.

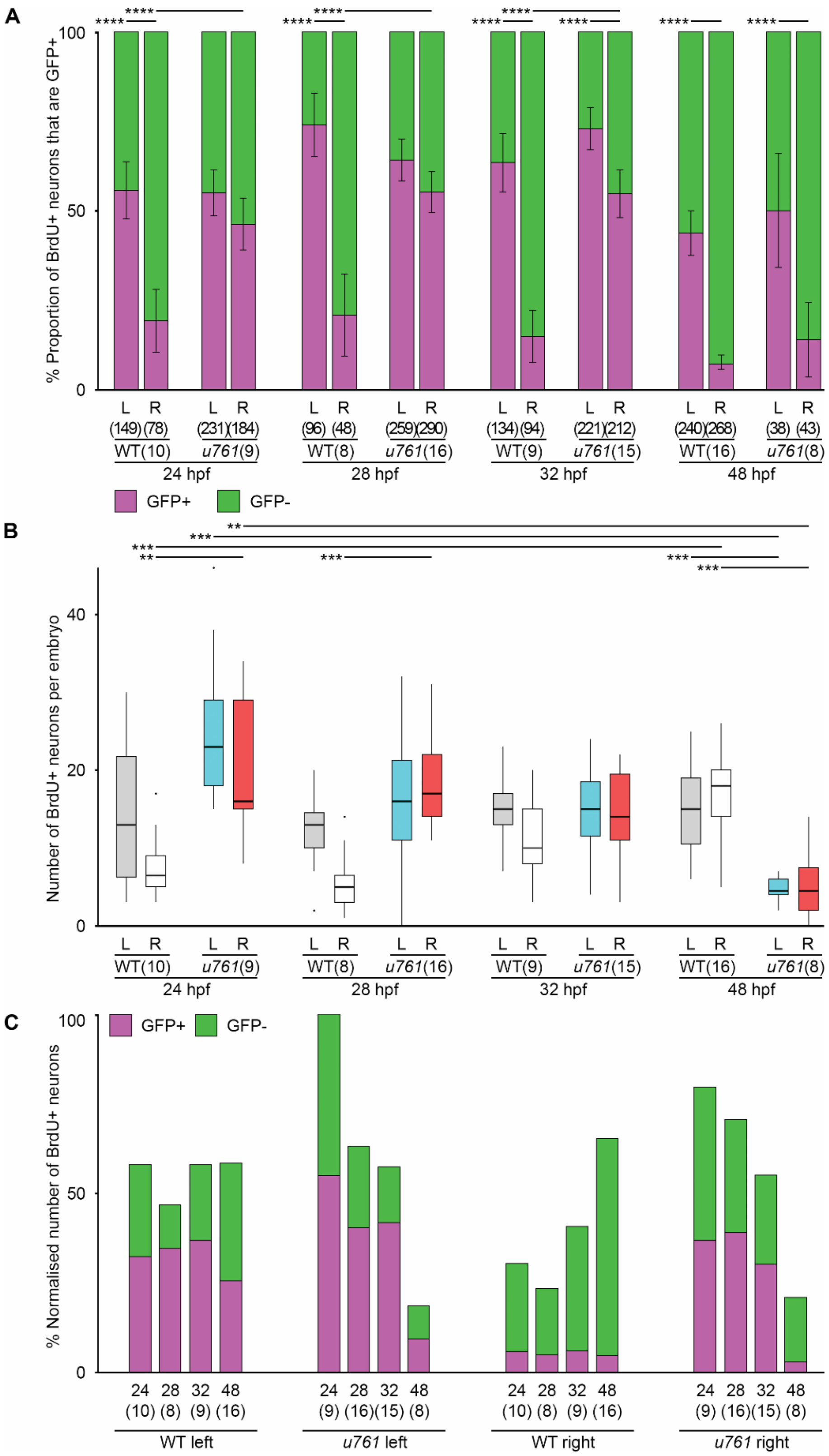

Fig. S11. Precocious neurogenesis in *cachd1*^{u761} mutant embryos with increased likelihood of acquiring lateral fate.

This is the author's version of the work. It is posted here by permission of the AAAS for personal use, not for redistribution. The definitive version was published in *Science* on 03/05/2024, DOI: 10.1126/science.ade6970.

(**A**) Bar chart showing the proportion of BrdU-positive cells that express the dHb_L marker transgene *pku588Et* (GFP+, magenta) in 5 dpf wildtype sibling and *cachd1*^{u761} mutants given a pulse of BrdU at different stages (24 hpf – 48 hpf). Note the change in proportion in the right habenula of *cachd1*^{u761} mutants, indicating an increased likelihood of acquiring dHb_L character. Error bars represent 95% confidence intervals of the proportion, calculated using a normal approximation, or Wilson score when the proportion was less than 0.1, and/or the number of larvae tested was less than 20. The total number of cells and larvae in each condition is indicated in brackets. Samples for 24 hpf are replicated from Fig. 2 for comparison. Q′ test of equality of proportions within each BrdU pulse timepoint only (degrees of freedom = 3; 24 hpf: $\chi^2 = 40.94$, $P = 6.7 \times 10^{-9}$; 28 hpf: $\chi^2 = 16.44$, $P = 9.2 \times 10^{-4}$; 32 hpf: $\chi^2 = 24.20$, $P = 2.3 \times 10^{-5}$; 48 hpf: $\chi^2 = 50.70$, $P = 5.7 \times 10^{-11}$) and *post hoc* pairwise comparisons using a modified Marascuilo procedure with Benjamini-Hochberg correction for multiple testing, * $0.1 \geq P > 0.05$, ** $0.05 \geq P > 0.01$, **** $P \leq 0.005$. (**B**) Boxplot showing the number of BrdU-positive cells in the habenulae of 5 dpf wildtype sibling (grey left, white right) and *cachd1*^{u761} mutants (cyan left, red right) given a pulse of BrdU at different stages (24 hpf – 48 hpf). Note the substantial increase in neurogenesis at early stages and decrease at later stages in *cachd1*^{u761} mutants. Although differences in neurogenesis between left and right habenulae in wildtype embryos were too small to detect with statistical significance in this study (for example, 28 hpf L vs R: $P = 0.1066$), an increase in neurogenesis in the right habenula was observed between 24 hpf and 48 hpf timepoints (24 hpf WT R vs 48 hpf WT R: $P = 0.0052$). The number of larvae in each condition is indicated in brackets. Kruskal-Wallis rank sum test (degrees of freedom = 15, $\chi^2 = 70.98$, $P = 3.0 \times 10^{-9}$) and *post hoc* pairwise comparisons using Wilcoxon rank sum test with continuity correction and Benjamini-Hochberg correction for multiple testing, * $0.1 \geq P > 0.05$, ** $0.05 \geq P > 0.01$, *** $0.01 \geq P > 0.005$, **** $P \leq 0.005$. A limited number of significant differences are presented here for clarity. (**C**) Summary bar chart showing the total number of BrdU-positive neurons observed in the habenula of 5 dpf in a wildtype and *cachd1*^{u761} mutant larvae labelled at each BrdU pulse timepoint that were expressing the dHb_L marker transgene *pku588Et* (GFP+, magenta) or not (GFP-, green), normalized to the highest number of observed labelled neurons and the number of larvae in each condition. Note for example, the increased proportion of GFP+ neurons born on the right-hand side of *cachd1*^{u761} mutants at early stages compared to WT (significant differences identified in A) and the increased total number of neurons (significant differences identified in B).

This is the author's version of the work. It is posted here by permission of the AAAS for personal use, not for redistribution. The definitive version was published in *Science* on 03/05/2024, DOI: 10.1126/science.ade6970.

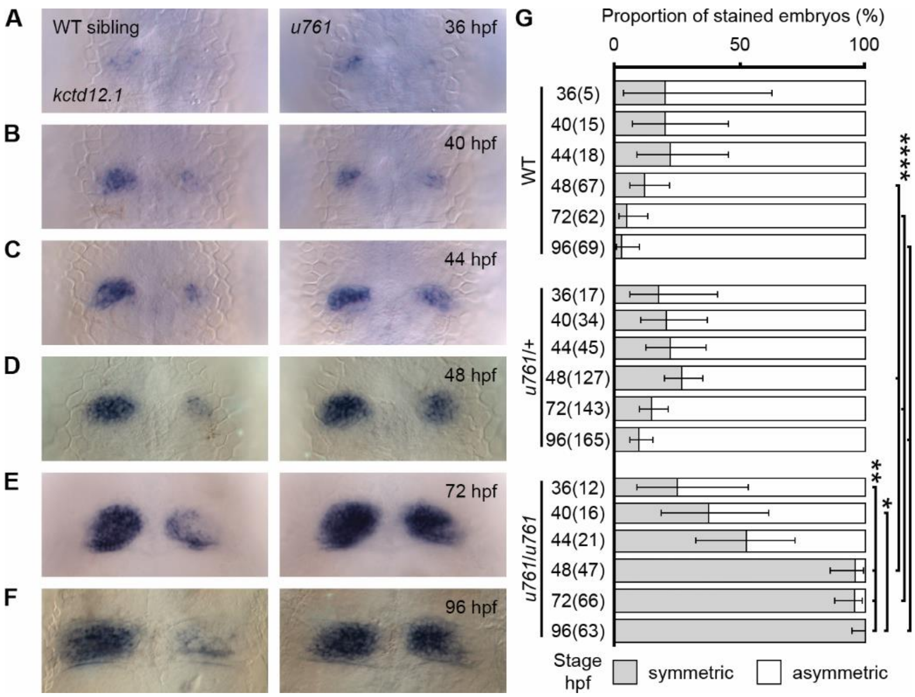

Fig. S12. Biased acquisition of left-sided character in right dorsal habenula of $cachd1^{u761}$ mutants.

(**A-F**) Dorsal views of wildtype sibling and $cachd1^{u761}$ mutant embryos at different developmental stages, 36 hpf (A) to 96 hpf (F), after colorimetric wholemount *in situ* hybridization with an antisense riboprobe for the dHb_L marker *kctd12.1*. Expression of *kctd12.1* is increased in the right habenula of $cachd1^{u761}$ mutants over the time course, consistent with an increased likelihood of acquiring dHb_L fate. (**G**) Bar chart showing the proportion of wildtype, *u761/+* and *u761/u761* mutant embryos that showed symmetric (grey) or overtly asymmetric (white) *kctd12.1* staining at the different stages. Error bars represent the 95% confidence intervals for the proportion calculated using the Wilson score. Number of embryos in each condition is indicated in brackets. Q′ test of equality of proportions (degrees of freedom = 17, χ^2 = 646.41, $P = 2.1 \times 10^{-126}$) and *post hoc* modified Marascuilo procedure for multiple comparisons of proportions with Benjamini-Hochberg correction for multiple testing was used to test significance, ** $0.05 \geq P > 0.01$, **** $P \leq 0.005$.

This is the author's version of the work. It is posted here by permission of the AAAS for personal use, not for redistribution. The definitive version was published in *Science* on 03/05/2024, DOI: 10.1126/science.ade6970.

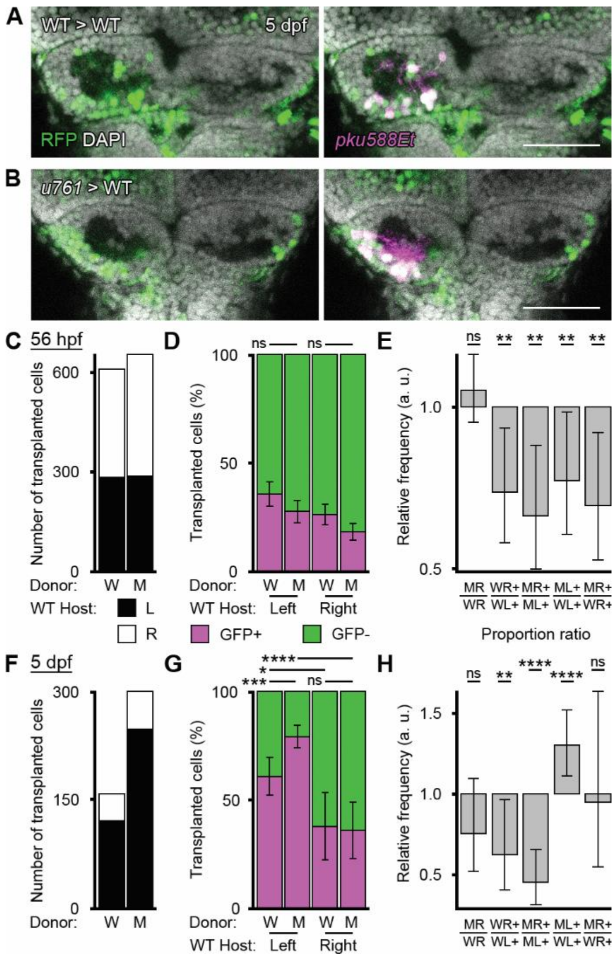

Fig. S13. Transplant experiments suggest that the loss of Cachd1 function does not have solely cell/lineage-autonomous consequences.

(**A**, **B**) Dorsal views of the habenulae of 5 dpf wildtype embryo hosts containing transplanted *pku588Et*, wildtype (A) or *u761* mutant (B) cells marked by expressing an RFP tracer (green). Expression of the *pku588Et* transgene (magenta) is specific to dHb_L cell types. Maximum projections of confocal stacks. Scale bars = 50 µm. (**C**, **F**) Charts showing the total number of transplanted *pku588Et*, wildtype (W) or *u761* mutant (M) cells counted in the left (black) or right (white) dorsal habenulae of 56 hpf (C) or 5 dpf (F) wildtype hosts (C: n = 25 (W), 31 (M); F: n = 4 (W), 12 (M)). (**D**, **G**) Charts showing the proportion of wildtype (W) or *u761* mutant (M) transplanted cells that express dHb_L-specific *pku588Et* (GFP+) in the left and right dorsal habenula of 56 hpf (D) or 5 dpf (G) WT hosts. Error bars represent the 95% confidence interval of the proportion. Q′ test of equality of proportions (D: degrees of freedom = 3, $\chi^2 = 21.04$, $P = 1.0 \times 10^{-4}$; G: degrees of freedom = 3, $\chi^2 = 48.47$, $P = 1.7 \times 10^{-10}$) and *post hoc* modified Marascuilo procedure with Benjamini & Hochberg correction for multiple testing. Only comparisons within transplant genotype or within position in host are shown for clarity. (**E**, **H**) Charts showing the relative frequency (risk) ratios of different proportions in the transplant data from 56 hpf (E) or 5 dpf (H) wildtype hosts. A value of 1 indicates equality of the proportions. Error bars represent the 95% confidence interval of the relative frequency. Fisher’s exact test with Benjamini-Hochberg correction for multiple testing. Note that there was an equal likelihood of a transplanted cell being observed in the left or right dorsal habenula regardless of genotype at both timepoints ($\hat{p}_{MR} \approx \hat{p}_{WR}$), although there were fewer transplanted cells on the right than left

This is the author's version of the work. It is posted here by permission of the AAAS for personal use, not for redistribution. The definitive version was published in *Science* on 03/05/2024, DOI: 10.1126/science.ade6970.

at 5 dpf. The likelihood of *pku588Et* expression was also asymmetric between left and right dorsal habenula for both wildtype and *u761* mutant cells at both timepoints ($\hat{p}_{WL+} > \hat{p}_{WR+}$ and $\hat{p}_{ML+} > \hat{p}_{MR+}$). At 56 hpf, the likelihood of a *u761* mutant transplant cell expressing *pku588Et* was reduced compared to wildtype cells on either side of the epithalamus ($\hat{p}_{WL+} > \hat{p}_{ML+}$ and $\hat{p}_{WR+} > \hat{p}_{MR+}$). However, at 5 dpf, *u761* mutant transplant cells were more likely to express *pku588Et* than wildtype cells in the left habenula ($\hat{p}_{WL+} < \hat{p}_{ML+}$) but equally likely in the right habenula ($\hat{p}_{WR+} \approx \hat{p}_{MR+}$). This suggests that the *u761* allele does not have strictly cell autonomous consequences on selection of subtype identity and that the environments of the left and right habenula are not equivalent. 'ns' $P > 0.1$, * $0.1 \geq P > 0.05$, ** $0.05 \geq P > 0.01$, *** $0.01 \geq P > 0.005$, **** $P \leq 0.005$.

This is the author's version of the work. It is posted here by permission of the AAAS for personal use, not for redistribution. The definitive version was published in *Science* on 03/05/2024, DOI: 10.1126/science.ade6970.

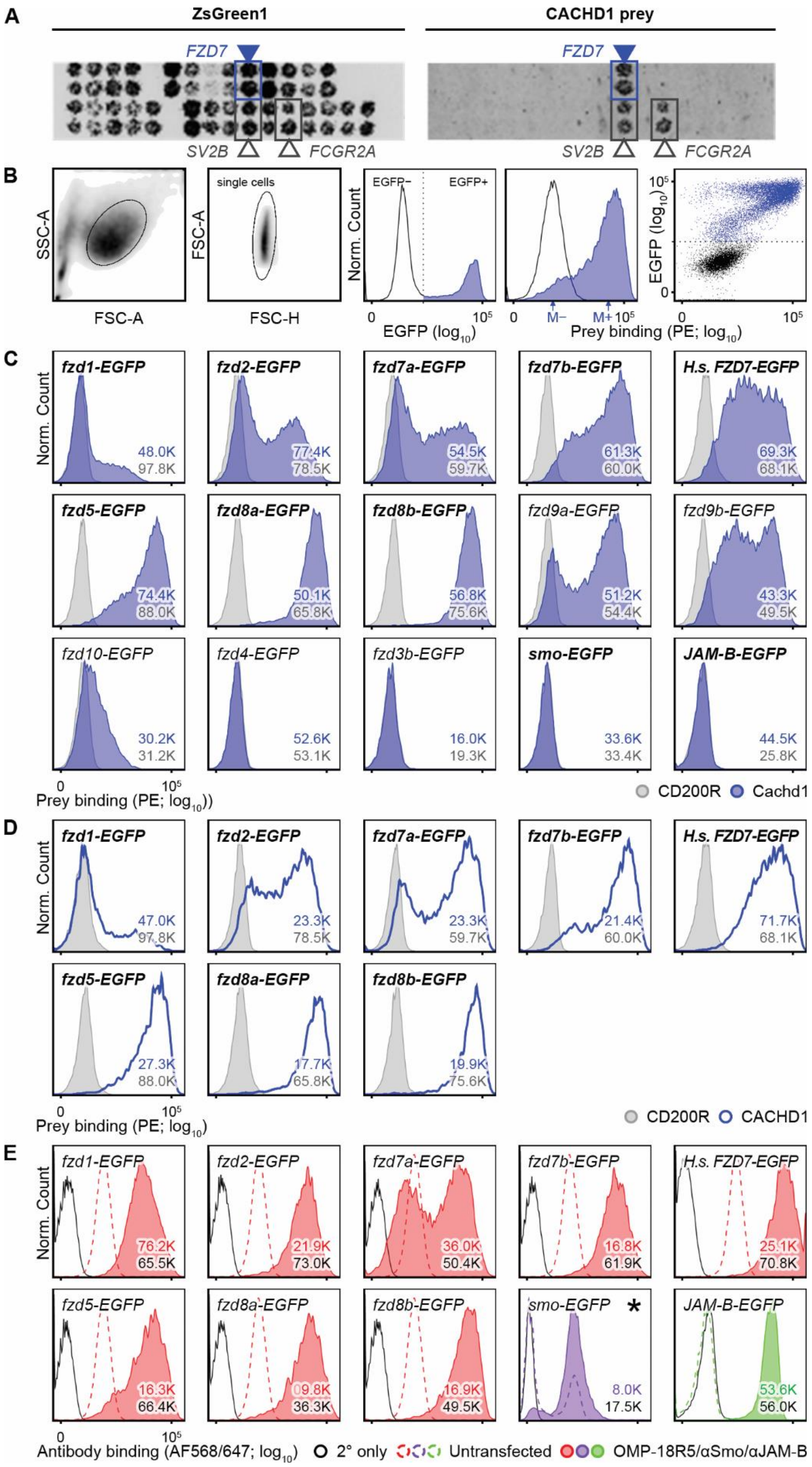

Fig. S14. Physical interactions of Cachd1 with Fzd family proteins detected by cell microarray and flow cytometry.

This is the author's version of the work. It is posted here by permission of the AAAS for personal use, not for redistribution. The definitive version was published in *Science* on 03/05/2024, DOI: 10.1126/science.ade6970.

(**A**) Cell microarray data showing the interaction between a human CACHD1 prey protein and its target FZD7 (blue). Expression vectors encoding both ZsGreen1 and FZD7, together with a range of interactions from alternative prey proteins were spotted onto slides, and human HEK293 cells were reverse transfected. Fixed cells were subsequently incubated with human CACHD1 prey protein and detected with an AlexaFluor-647 conjugated secondary antibody. ZsGreen fluorescence was used to confirm transfection efficiency and spot locations on the slides (left-hand panel). Two other hits, SV2B and FCGR2A, were considered false positives because of multiple binding interactions with a wide range of prey proteins. (**B**) Gating strategy for testing specific interactions with transiently transfected cells. Single cells were isolated by forward (FSC-A, FSC-H) and side (SSC-A) light scatter, then separated into EGFP-negative (untransfected or not expressing the EGFP fusion protein bait; black outline) and EGFP-positive (transfected, blue) gates. Prey binding is indicated by increased PE fluorescence in the EGFP-positive population. The ratio of median PE fluorescence of either subpopulation ($\Delta M_{PE} = \ln(M_{PE}^{EGFP+}/M_{PE}^{EGFP-})$) was used to quantify the degree of prey binding to the EGFP-positive population. (**C**, **D**) Examples of normalized histograms of EGFP-positive populations for each EGFP fusion protein bait transfection indicated, tested with either zebrafish Cachd1 (C, solid blue), human CACHD1 (D, blue outline) or CD200R negative control prey (grey). Note that human CACHD1 prey is able to bind zebrafish Fzd family proteins and *vice versa*, suggesting conservation of binding (not all combinations tested). Numbers in each panel indicate the total number of EGFP-positive events collected for each condition over all replicates. Bold transfection titles indicate validation of surface expression with antibodies. (**E**) Examples of normalized histograms of EGFP-positive populations for each EGFP fusion protein bait tested with antibodies to detect surface expression of Fzd family proteins (OMP-18R5, red), Smo-EGFP (purple) or negative control bait protein JAM-B-EGFP (green). The dotted line in each panel indicates antibody binding fluorescence in mock transfected HEK293E cells; black outline indicates the secondary only negative control. Note that untransfected HEK293E cells appear to have endogenous surface expression of Fzd family receptor(s) and the Smo receptor (dotted lines). Numbers in each panel indicate the total number of EGFP-positive events collected for each condition. Asterisk indicates a formaldehyde-fixed cell population.

This is the author's version of the work. It is posted here by permission of the AAAS for personal use, not for redistribution. The definitive version was published in *Science* on 03/05/2024, DOI: 10.1126/science.ade6970.

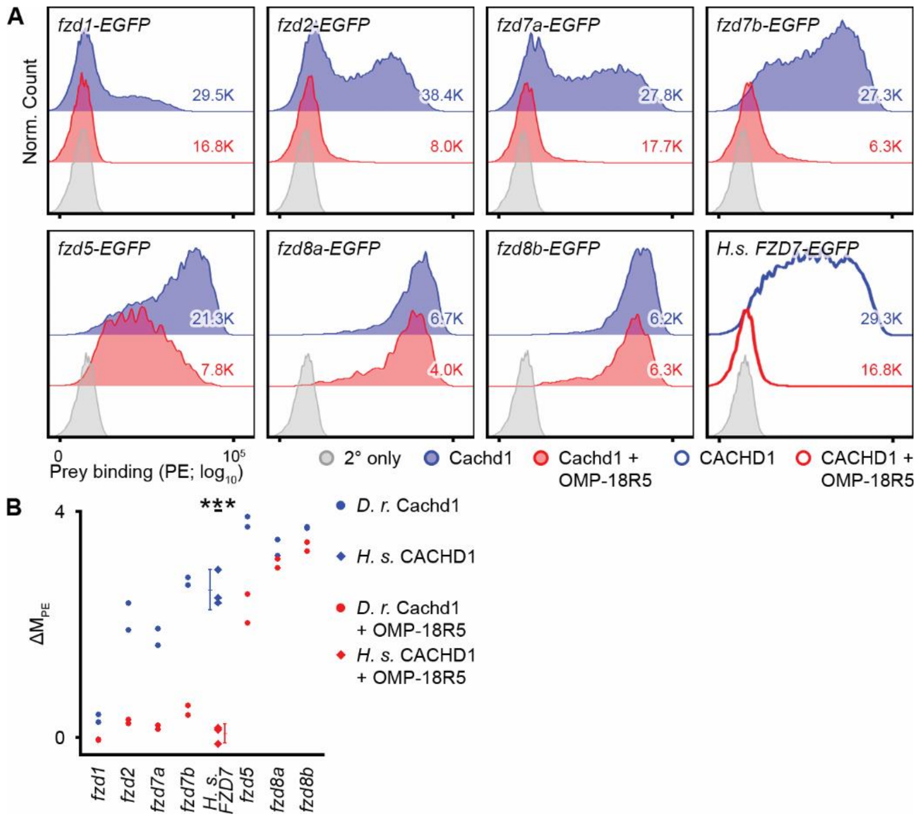

Fig. S15. Cachd1 prey binding to Fzd family receptors blocked by anti-Fzd antibody. (**A**) Examples of normalized histograms of EGFP-positive populations for each EGFP fusion protein bait transfection indicated, tested with either zebrafish Cachd1 prey protein alone (blue) or after pre-incubation of the cells with OMP-18R5 antibody (red). Human FZD7-EGFP histogram duplicated from Fig. 3A for comparison. Secondary antibody only control shown in grey. Total number of events collected for each condition indicated in each plot. Note that Cachd1 prey binding to zebrafish Fzd family bait proteins Fzd1/2/7a/7b is effectively blocked by OMP-18R5 pre-incubation, but not to Fzd5/8a/8b. (**B**) Dot plot showing ΔM_{PE} for transfections tested for Cachd1 prey protein interaction (circles and diamonds represent zebrafish and human prey proteins respectively) with (red) or without pre-incubation with OMP-18R5 (blue). Mean indicated with a single line, error bars indicate 95% confidence intervals of the mean. One-tailed paired *t*-test for human CACHD1-FZD7 interaction only (degrees of freedom = 2, $t = 9.53$, *** $P = 0.0054$), due to limited numbers of replicates for other inhibition tests.

This is the author's version of the work. It is posted here by permission of the AAAS for personal use, not for redistribution. The definitive version was published in *Science* on 03/05/2024, DOI: 10.1126/science.ade6970.

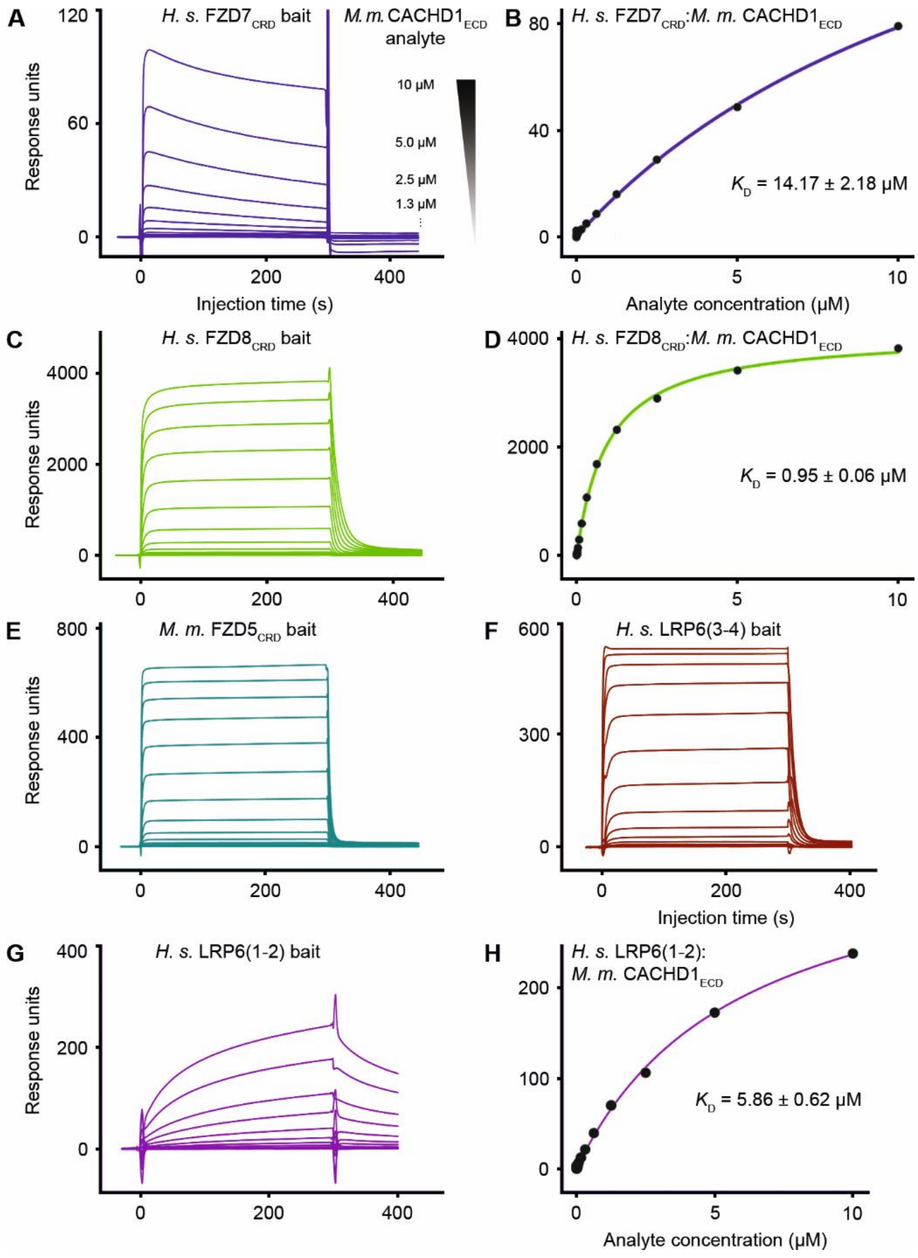

Fig. S16. SPR analysis of CACHD1$_{ECD}$ interactions with LRP6 and FZD$_{CRD}$ domains. (**A**, **C**, **E**-**G**) Surface plasmon resonance sensorgrams showing the response of different concentrations of mouse CACHD1$_{ECD}$ analyte flowing over surfaces of immobilized human FZD7$_{CRD}$ (A), human FZD8$_{CRD}$ (C), mouse FZD5$_{CRD}$ (E), human LRP6$_{P3E3P4E4}$ (3-4, F), and LRP6$_{P1E1P2E2}$ (1-2, G). (**B**, **D** and **H**) Graphs showing the determination of the equilibrium constants (K_D ± 95% C.I.) for those interactions, except for FZD5$_{CRD}$ and LRP6$_{P3E3P4E4}$ which are shown in Figure 3.

This is the author's version of the work. It is posted here by permission of the AAAS for personal use, not for redistribution. The definitive version was published in *Science* on 03/05/2024, DOI: 10.1126/science.ade6970.

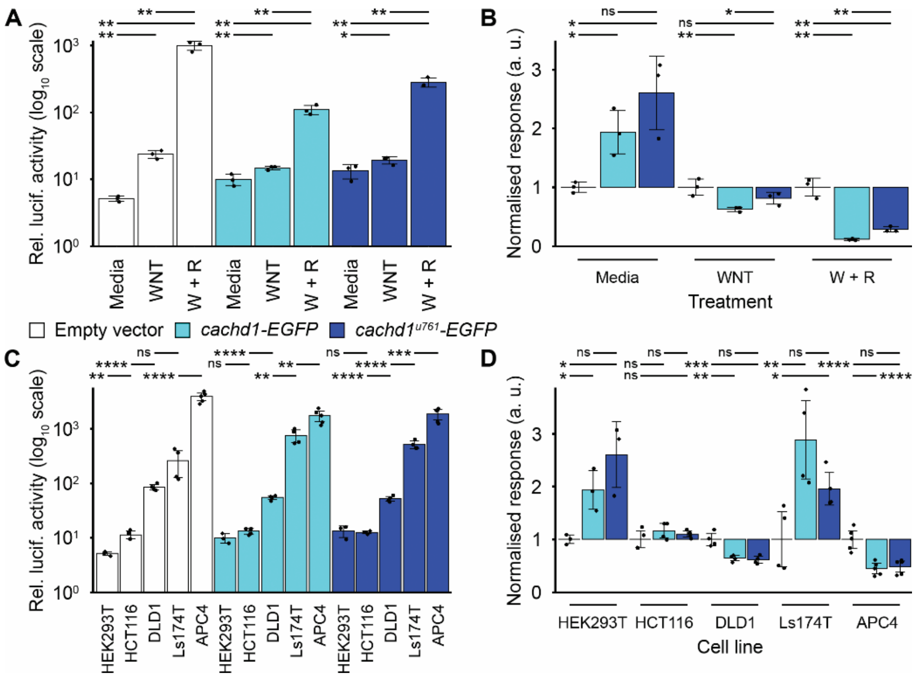

Fig. S17. Overexpression of Cachd1 strongly antagonizes RSPONDIN1-based sensitivity to canonical Wnt signaling and modulates pathway activity.

(**A-D**) Summary charts of TOP-FLASH assays performed in HEK293T (A and B) and cancer cell lines (C and D, HEK293T data from B replicated for comparison) transfected with zebrafish *cachd1-EGFP* (cyan), *cachd1^{u761}-EGFP* (dark blue) or empty vector control (white). (A) Mean relative luciferase responses (log_{10} scale) of transfected HEK293T cells after treatment with media alone (Media), WNT3A-conditioned media (WNT) or WNT3A and RSPONDIN1-condition media (W + R). (B) Responses in (A) normalized to the empty vector control for each treatment. (C) Mean relative luciferase responses (log_{10} scale) of transfected HEK293T and Wnt pathway mutant cancer cell lines HCT116, DLD1, Ls174T and APC4, in media alone. (D) Responses in (C) normalized to the empty vector control for each cell type. Note that transfection of cells with the *cachd1^{u761}* mutant construct also elicited effects on canonical Wnt signaling responses, to a lesser degree than wildtype. This is most likely an overexpression artefact, as high expression leads to the presence of mutant Cachd1 protein on the cell surface (see Fig. S1A). Individual points represent the mean response of an individual experiment, calculated from at least triplicate measurements, and error bars indicate 95% confidence intervals of the mean. One way Welch test of means were performed on raw response data (not assuming equal variances; A: $F = 53.25$, D. $F_{num} = 8.00$, D. $F_{denom} = 7.05$, $P = 1.3 \times 10^{-5}$; C: $F = 65.01$, D. $F_{num} = 14.00$, D. $F_{denom} = 16.33$, $P = 1.4 \times 10^{-11}$), *post hoc* pairwise *t*-tests with non-pooled standard deviations, Benjamini-Hochberg correction for multiple testing; only significant differences within transfection group (A, C), treatment group (B) or cell line (D) are presented here for clarity, ‘ns’ $P > 0.1$, * $0.1 \geq P > 0.05$, ** $0.05 \geq P > 0.01$, *** $0.01 \geq P > 0.005$, **** $P \leq 0.005$.

This is the author's version of the work. It is posted here by permission of the AAAS for personal use, not for redistribution. The definitive version was published in *Science* on 03/05/2024, DOI: 10.1126/science.ade6970.

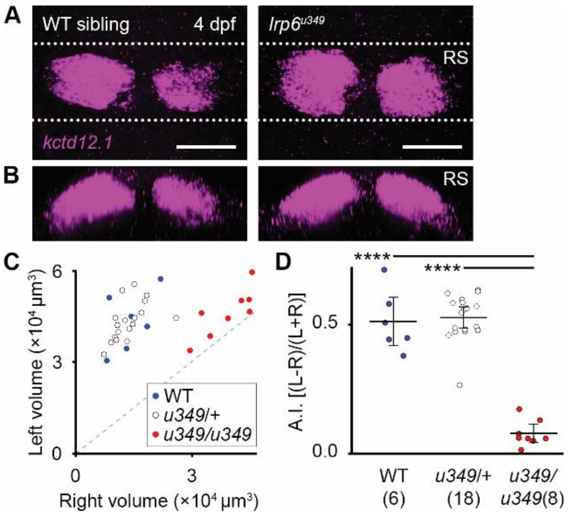

Fig. S18. Quantification of *lrp6* mutant phenotype.
(**A, B**) Dorsal (A) and (B) transverse projection of 4 dpf wildtype sibling and *lrp6*^{u349} mutant larvae after fluorescent RNA *in situ* hybridization with riboprobes against *kctd12.1*. Maximum projections of confocal stacks. Dotted lines represent the approximate volume in the transverse projections (RS, B). Scale bars = 50 µm. (**C**) Scatterplot showing quantification of *kctd12.1*-fluorescent volumes in the left and right habenulae of wildtype (blue), *u349/+* (white) and *u349/u349* (red) siblings at 4 dpf, represented with a single point. The grey dashed line represents the line of symmetry between left and right volumes. Note the increase in the volume of *kctd12.1* in the right habenula of *lrp6*^{u349} mutants compared to wildtype or heterozygous siblings. (**D**) Dot plot showing the asymmetry index calculated using *kctd12.1* volumes for each wildtype, *u349/+* and *u349/u349* sibling larvae. Mean asymmetry index for each genotype is indicated with a horizontal bar. Error bars represent 95% confidence intervals of the mean. Number of larvae for each condition indicated in brackets. Wildtype and *lrp6*^{u349} heterozygous siblings have leftward asymmetry of the *kctd12.1* marker, but the increase in right habenula volume renders *lrp6*^{u349} mutants symmetric. ANOVA (degrees of freedom = 2, F = 77.34, P = 2.4 × 10^{-12}) and *post hoc* Tukey pairwise comparisons were used for hypothesis testing, **** $P \leq$ 0.005.

This is the author's version of the work. It is posted here by permission of the AAAS for personal use, not for redistribution. The definitive version was published in *Science* on 03/05/2024, DOI: 10.1126/science.ade6970.

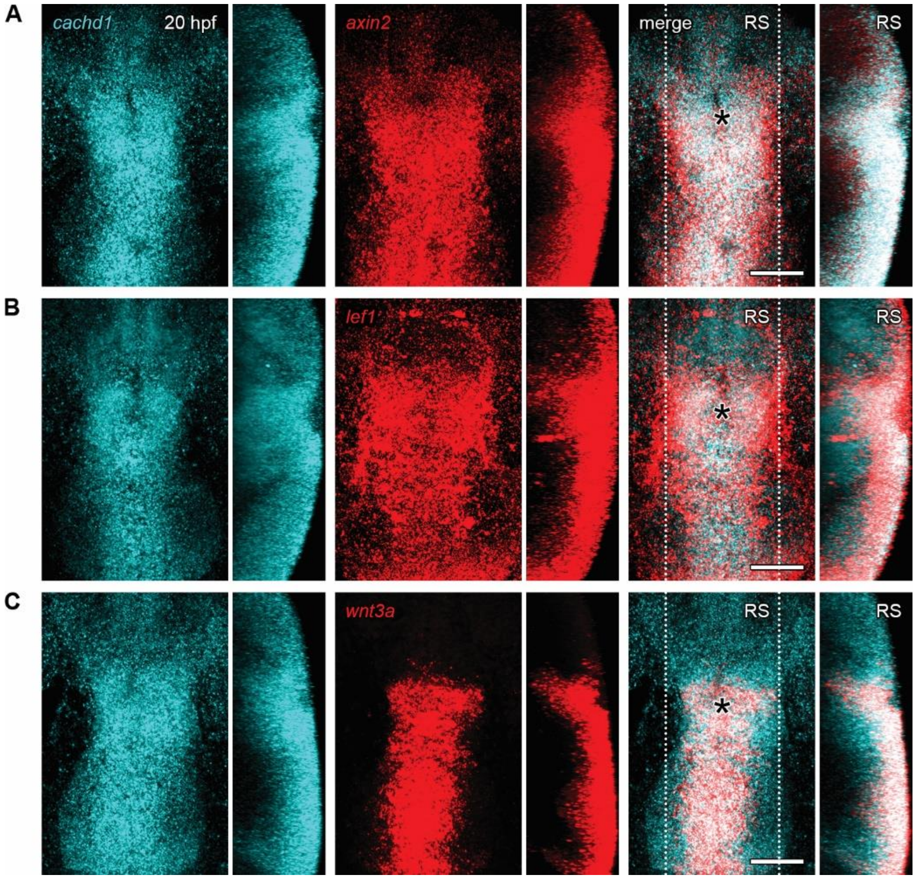

Fig. S19. *cachd1* is co-expressed with other Wnt pathway genes in the dorsal diencephalon and midbrain roof plate.
(**A-C**) Dorsal views (left panels) and sagittal projections (RS, right panels) of 20 hpf wildtype embryos after double fluorescent *in situ* hybridization staining with antisense riboprobes for *cachd1* (cyan) and Wnt pathway genes *axin2* (A, red), *lef1* (B, red) or *wnt3a* (C, red) showing *cachd1* is expressed in Wnt active tissues in early development. The approximate position of the pineal is marked with an asterisk. Maximum projections of confocal stacks. Dotted lines represent the approximate volume shown in the sagittal projections (RS). Scale bars = 50 µm.

This is the author's version of the work. It is posted here by permission of the AAAS for personal use, not for redistribution. The definitive version was published in *Science* on 03/05/2024, DOI: 10.1126/science.ade6970.

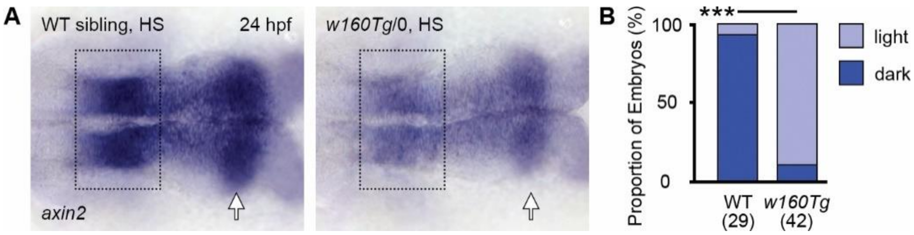

Fig. S20. Heat shock induction of Cachd1 reduces expression of *axin2*.
(**A**) Representative dorsal views of flat mounted wildtype (left) and *Tg(HSE:cachd1, EGFP)w160* (right) 24 hpf embryos, heat shocked at 18-20 somites stage, after *in situ* hybridization with an *axin2* riboprobe (anterior left). The dorsal diencephalon is indicated with a dotted box and the midbrain-hindbrain boundary with a white arrow. Note the decrease in staining intensity in the *w160Tg* hemizygote embryos. (**B**) Chart showing the proportion of heat shocked embryos with dark or light *axin2* staining (subjectively assessed). Numbers in brackets indicate the total number of embryos tested. Fisher’s exact test, *** $P < 0.01$.

This is the author's version of the work. It is posted here by permission of the AAAS for personal use, not for redistribution. The definitive version was published in *Science* on 03/05/2024, DOI: 10.1126/science.ade6970.

Table S1. Cross validation of anti-Cachd1 antibody and *cachd1* null allele.

Genotype	IHC Staining Intensity			Total
	High	Low	Absent	
wildtype	11	2	0	13
sa17010/+	0	19	0	19
sa17010/*sa17010*	0	1	10	11
n. d.	0	1	0	1
Total	11	23	10	44

Fisher's exact test: $P = 4.0 \times 10^{-7}$

This is the author's version of the work. It is posted here by permission of the AAAS for personal use, not for redistribution. The definitive version was published in *Science* on 03/05/2024, DOI: 10.1126/science.ade6970.

Table S2. Data collection and refinement statistics for the $CACHD1_{ECD}$:$FZD5_{CRD}$:LRP6 $_{P3E3P4E4}$ ternary complex (PDB:8S7C).

Data collection		Refinement	
Source	Diamond I03	Resolution (Å)	72.33-4.72
Wavelength(Å)	0.9762	No. unique reflections	23489(154)
Space group	$C2_1$	R_{work} / R_{free}	0.196/0.243
Cell dimensions:		No. atoms:	41757
a, b, c (Å)	283.70, 198.24, 218.82	Protein	41211
α, β, γ (°)	90, 128.08, 90	Ligand/ion	546
Resolution (Å)	172.26-4.72(4.87) *	Water	0
R_{sym} or R_{merge}	0.24(---)	*B*-factors:	
$I / \sigma I$	6.3(1.6)	Protein	211.42
Completeness (%)	88.8(68.0)	Ligand/ion	216.30
Redundancy	6.8(7.0)	Water	n/a
CC(1/2)	0.99(0.44)	R. m. s. deviations:	
		Bond lengths (Å)	0.004
		Bond angles (°)	0.86

* Values in parentheses are for highest-resolution shell.

This is the author's version of the work. It is posted here by permission of the AAAS for personal use, not for redistribution. The definitive version was published in *Science* on 03/05/2024, DOI: 10.1126/science.ade6970.

Table S3. Allelic series of *lrp6* nonsense mutations show a bilateral 'double left' phenotype.

lrp6 allele number	Genomic lesion (Zv11 assembly)	Freq. of double left phenotype*				χ^2 (2 d. o. f.)	*P* value
		WT	+/-	-/-	Total		
u348	11 bp del. + 9 bp del. (4:76249-76259del; 76390-76398del, p.L45RfsX28)	0/27	2/44	27/29	100	81.7	1.8×10^{-18}
u349	4 bp ins. (4:76250-76251insATGG, p.V46MfsX32)	0/17	0/55	18/19	91	n. a. †	8.0×10^{-18}
u350	23 bp del. (4:76390-76412del, p.V92GfsX11)	0/25	0/46	26/27	98	93.1	6.2×10^{-21}
u351	149 bp del. + 9 bp ins. (4:76242-76390del; insCACGCCACG, p.A43HfsX21)	0/12	0/16	9/9	37	n. a. †	8.0×10^{-9}

* assessed by colorimetric *in situ* hybridisation with *kctd12.1* riboprobe

† Fisher's exact test was used where expected values were below 5

This is the author's version of the work. It is posted here by permission of the AAAS for personal use, not for redistribution. The definitive version was published in *Science* on 03/05/2024, DOI: 10.1126/science.ade6970.

Table S4. Oligonucleotides for generating CRISPR/Cas9 sgRNAs targeting *lrp6* exon 2.

Gene	Guide	Primer sequence (target sequence underlined in bold)
lrp6	sg1	GCGTAATACGACTCACTATA**GGCCAACGCCACGCTGGTGA**GTTTTAGAGCTAGAAATAGCAAG
lrp6	sg2	GCGTAATACGACTCACTATA**GGCCAGACCGGAGATGACGG**GTTTTAGAGCTAGAAATAGCAAG
Template oligo sequence:		AAAAGCACCGACTCGGTGCCACTTTTTCAAGTTGATAACGGACTAGCCTTATTTTAACTTGCTATTTCTAGCTCTAAAAC

This is the author's version of the work. It is posted here by permission of the AAAS for personal use, not for redistribution. The definitive version was published in *Science* on 03/05/2024, DOI: 10.1126/science.ade6970.

Table S5. Primer sequences used for mapping, genotyping, qPCR, headloop PCR and cloning.

Species	Gene	Name	Sequence
Zebrafish	*ak4*	e1-Mapping-F	CTGTTTTGCACCTCCAACCT
Zebrafish	*ak4*	e1-Mapping-R	GCTTCACGGAGCATATGACA
Zebrafish	*cachd1*	i8-9-Mapping-F	TTTCAACACTTTGGCCTGTT
Zebrafish	*cachd1*	i8-9-Mapping-R	GCAGTGCAGAAGAGGGTTTC
Zebrafish	*cachd1*	u761-AloI-F	TTTAACTGCACTGTTTTGCCTTA
Zebrafish	*cachd1*	u761-AloI-R	ATAGGCATAAACGGCGAACA
Zebrafish	*cachd1*	HRMA-sa17010-F	GAGCAATCTGGAGCTGGGTT
Zebrafish	*cachd1*	HRMA-sa17010-R	TGTATGTCGCGGCAGTAAGG
Zebrafish	*cachd1*	cloning-FL-SalI-F	ATGAACGTCGACTGCGAAACGGAAAAG TTAGG
Zebrafish	*cachd1*	cloning-FL-SacII-R	TGCAATCCGCGGGCACTCAGCGTCCAC ACT
Zebrafish	*cachd1*	RT-PCR-e1-F*	TGCGAAACGGAAAAGTTAGG
Zebrafish	*cachd1*	RT-PCR-e9-R	CCCACTGTGGTCTCCAGATT
Zebrafish	*cachd1*	RT-PCR-e8-F	CGCAGTGAAAGAGGAGAACC
Zebrafish	*cachd1*	RT-PCR-e17-R	CCGAATTTTGTTGCCTTTGT
Zebrafish	*cachd1*	RT-PCR-e16-F	CTGGACCGTACCTGGATGTT
Zebrafish	*cachd1*	RT-PCR-e27-R*	TGAGGGTTGGTTCTGAGGTC
Zebrafish	*lrp6*	CRISPR-sg1-HRMA-F	TGTGTTCCACTGGAGTGACATT
Zebrafish	*lrp6*	CRISPR-sg1-HRMA-R	CAGGCCCTGCGCATAGATATAA
Zebrafish	*lrp6*	CRISPR-sg2-HRMA-F	GATCTACTGGAGCGACGTGAG
Zebrafish	*lrp6*	CRISPR-sg2-HRMA-R	TCGGAGTCGGTCCAGTAAAGTT
Zebrafish	*lrp6*	HLPCR-control-F	AGATGTTTTGAAGAGTGCGGTG
Zebrafish	*lrp6*	HLPCR-control-R	TAAACATCCCGAAAACAAGCTGC
Zebrafish	*lrp6*	HLPCR-HL-sg1-F	CCATCACCAGCGTGGCGTTGAGATGTT TTGAAGAGTGCGGTG

This is the author's version of the work. It is posted here by permission of the AAAS for personal use, not for redistribution. The definitive version was published in *Science* on 03/05/2024, DOI: 10.1126/science.ade6970.

Zebrafish	*lrp6*	HLPCR-HL-sg2-R	CACCGTCATCTCCGGTCTGGTAAACAT CCCGAAAACAAGCTGC
Zebrafish	*slc18a3b*	riboprobe-cloning-F	GGAGAGCTCGTGCGTAATTC
Zebrafish	*slc18a3b*	riboprobe-cloning-R	CACTTAGAGGCGTCCATCGT
Zebrafish	*zgc:101731*	riboprobe-cloning-F	GGTGTTGAGCGAGAGTTGGT
Zebrafish	*zgc:101731*	riboprobe-cloning-R	TGTTTTCAAACCTTTGACTGG
Zebrafish	*aoc1*	riboprobe-cloning-F	ACAACGGGCAGTATTTCGAC
Zebrafish	*aoc1*	riboprobe-cloning-R	TTCTGTAGCGCACAGGTTTG
Zebrafish	*kiss1*	riboprobe-transcription-F	ATGCTGCTTACTGTCATATTGATG
Zebrafish	*kiss1*	riboprobe-T3-transcription-R	GGATCCATTAACCCTCACTAAAGGGAC ACCTAAAACATGAAGGCAAATACC
Zebrafish	*fzd1*	fusion-PCR-5'-F	TGGCCGACTGGAGACTCTTTC
Zebrafish	*fzd1*	fusion-PCR-5'-R	CACTGGAAGCGTCCGCGCTG
Zebrafish	*fzd1*	fusion-PCR-3'-F	CTCCCAGCGCGGACGCTTCC
Zebrafish	*fzd1*	fusion-PCR-3'-R	CGAAAGCAGAGCTTCACACTGTGG
Zebrafish	*fzd1*	cloning-NotI-F	GCGGCCGCCACCATGGCAGCTCGCGCT CTCTTC
Zebrafish	*fzd1*	cloning-AscI-R	GACTATGGCGCGCCCACTGTGGTTTCT CCCTGTTTGCTGTTCGCC
Zebrafish	*fzd2*	cloning-NotI-F	GCGGCCGCCACCATGGCAGCGAGTGGA AGTGTG
Zebrafish	*fzd2*	cloning-AscI-R	GAGTGAGGCGCGCCAACAGTGGTTTCT CCTTGTCC
Zebrafish	*fzd3b*	cloning-NotI-F	GCGGCCGCCACCATGGGCTGTGTTGTG GATTTACC
Zebrafish	*fzd3b*	cloning-AscI-R	TAGTATGGCGCGCCCGCACTGGTCCCG TTCTCCGG
Zebrafish	*fzd4*	cloning-NotI-F	GCGGCCGCCACCATGGCTCGGTTTGAG TTCGGG
Zebrafish	*fzd4*	cloning-AscI-R	TAGTTAGGCGCGCCCACAACCGTCTCG TTTCCTTTGCCCGG
Zebrafish	*fzd5*	cloning-NotI-F	GCGGCCGCCACCATGGGGAAACCTGCA GACGAG
Zebrafish	*fzd5*	cloning-AscI-R	GACGCTGGCGCGCCGACATGTGATGAG GGTGCTGATTTGTG

This is the author's version of the work. It is posted here by permission of the AAAS for personal use, not for redistribution. The definitive version was published in *Science* on 03/05/2024, DOI: 10.1126/science.ade6970.

Zebrafish	*fzd7a*	cloning-NotI-F	GCGGCCGCCACCATGGCTTTCCTCAAGATGCAAC
Zebrafish	*fzd7a*	cloning-AscI-R	TAGTATGGCGCGCCTACCGTCGTCTCGCCCTGGT
Zebrafish	*fzd7b*	cloning-NotI-F	GCGGCCGCCACCATGGCGGTACGGGAAGTTGG
Zebrafish	*fzd7b*	cloning-AscI-R	GAGTGAGGCGCGCCCACCGTTGTTTCCCCTTGGTTG
Zebrafish	*fzd8a*	cloning-NotI-F	GCGGCCGCCACCATGGAGTGCTACCTGTTGGG
Zebrafish	*fzd8a*	cloning-AscI-R	TACTCTGGCGCGCCGACTTGGGACAAAGGCATCTGCTTGGG
Zebrafish	*fzd8b*	fusion-PCR-5'-F	CCAGAGCACATGCCAGCGCATCC
Zebrafish	*fzd8b*	fusion-PCR-5'-R	GTGCACAGGGCTCGCCAGGAATC
Zebrafish	*fzd8b*	fusion-PCR-3'-F	GATTCCTGGCGAGCCCTGTGCAC
Zebrafish	*fzd8b*	fusion-PCR-3'-R	TCATCACACACGAGAAAGTGGCATTTGTTTTGGAGG
Zebrafish	*fzd8b*	cloning-NotI-F	GCGGCCGCCACCATGGACTCGCCTACACAGGG
Zebrafish	*fzd8b*	cloning-AscI-R	GACGCTGGCGCGCCCACACGAGAAAGTGGCATTTGTTTTGG
Zebrafish	*fzd9a*	cloning-NotI-F	GCGGCCGCCACCATGGGACATTGCATGAAGATTGGG
Zebrafish	*fzd9a*	cloning-AscI-R	TGATCGGGCGCGCCAACATGTGTGGGACTGTCTGTATAG
Zebrafish	*fzd9b*	cloning-NotI-F	GCGGCCGCCACCATGGGAAGCTCACCTCTGCAAATTG
Zebrafish	*fzd9b*	cloning-AscI-R	TAGCTCGGCGCGCCTACATGTGTGGGACAGTCTGAGTAGG
Zebrafish	*fzd10*	cloning-NotI-F	GCGGCCGCCACCATGGTTGCTGCCGGTGTCGG
Zebrafish	*fzd10*	cloning-AscI-R	GAGCGTGGCGCGCCTACACAAGTTGCAGGAGGACCTGCTG
Zebrafish	*smo*	cloning-NotI-F	GCGGCCGCCACCATGTCCTCCAAGCGCCCCTGCTCCATT
Zebrafish	*smo*	cloning-AscI-R	TGCGCAGGCGCGCCAAAATCTGAGTCAGCATCCAATAGCTCAGC

This is the author's version of the work. It is posted here by permission of the AAAS for personal use, not for redistribution. The definitive version was published in *Science* on 03/05/2024, DOI: 10.1126/science.ade6970.

Zebrafish	*gng8*	cloning-PCR-F	CATCATACTAGTGGGCTATAAAACAAAATG
Zebrafish	*gng8*	cloning-PCR-R	CATCATGATATCTTCGTTTGTAGAGACCAA
Mouse	*Cachd1*	qPCR-F	AGTTCAGCAGCTAGCCAAAAA
Mouse	*Cachd1*	qPCR-R	CCATCAAACTCCATCATGGA
Mouse	*Ccnd1*	qPCR-F	GCCATCCAAACTGAGGAAAA
Mouse	*Ccnd1*	qPCR-R	GATCCTGGGAGTCATCGGTA
Mouse	*Axin2*	qPCR-F	TCCAGAGAGAGATGCATCGC
Mouse	*Axin2*	qPCR-R	AGCCGCTCCTCCAGACTATG
Mouse	*Hprt1*	qPCR-F	TCATGAAGGAGATGGGAGGC
Mouse	*Hprt1*	qPCR-R	CCACCAATAACTTTTATGTCCCC
Human	*CACHD1*	qPCR-F	CTTAAATTCAGTTCTTGCAG
Human	*CACHD1*	qPCR-R	CGTAGATGGGTCTACTGCGG
Human	*CCND1*	qPCR-F	CTCCGCCTCTGGCATTTTGG
Human	*CCND1*	qPCR-R	TCTCCTTGCAGCTGCTTAG
Human	*AXIN2*	qPCR-F	AGTGTGAGGTCCACGGAAAC
Human	*AXIN2*	qPCR-R	CTTCACACTGCGATGCATTT
Human	*ACTB*	qPCR-F	TTCTACAATGAGCTGCGTGTG
Human	*ACTB*	qPCR-R	GGGGTGTTGAAGGTCTCAAA
Human	*FZD7*	cloning-NotI-F	GCGGCCGCCACCATGCGAGACCCAGGTGCAG
Human	*FZD7*	cloning-AscI-R	GAGTGAGGCGCGCCTACCGCAGTCTCCCCCTTGC
Jellyfish	***EGFP***	cloning-AscI-F	TAGTATGGCGCGCCGGGTAGCAAGGGCGAGGAGC
Jellyfish	***EGFP***	cloning-BamHI-R	GAGGCAGGATCCTCACTTGTACAGCTCGTCCATGCCG

* also used for riboprobe cloning of *cachd1*

This is the author's version of the work. It is posted here by permission of the AAAS for personal use, not for redistribution. The definitive version was published in *Science* on 03/05/2024, DOI: 10.1126/science.ade6970.

Table S6. Source of plasmids used as templates for *smo*- and *fzd-EGFP* flow cytometry, SPR and crystallography constructs.

Species	Gene	Construct ID	Source	Vector
Zebrafish	*fzd1*	IMAGE 9038402	Source Biosciences	pCR4-TOPO
Zebrafish	*fzd2*		Prof. Masa Tada (gift) *(80)*	
Zebrafish	*fzd3b*	IMAGE 7040422	Source Biosciences	pExpress-1
Zebrafish	*fzd4*	Synthesized clone ODa20912: XM_005173425	GenScript	pcDNA3.1+-DYK
Zebrafish	*fzd5*	IMAGE 9037464	Source Biosciences	pCR4-TOPO
Zebrafish	*fzd6*	IMAGE 6971142	Source Biosciences	pCMV-SPORT6.1
Zebrafish	*fzd7a*		Prof. Masa Tada (gift) *(81)*	
Zebrafish	*fzd7b*	IMAGE 5777452	Source Biosciences	pME18S-FL3
Zebrafish	*fzd8a*	IMAGE 7002555	Source Biosciences	pExpress-1
Zebrafish	*fzd8b*	IMAGE 6802128	Source Biosciences	pCMV-SPORT6.1
Zebrafish	*fzd9a*	Synthesized clone ODa11014: XM_003198686	GenScript	pcDNA3.1+-DYK
Zebrafish	*fzd9b*	IMAGE 9038534	Source Biosciences	pCR4-TOPO
Zebrafish	*fzd10*	IMAGE 7042011	Source Biosciences	pExpress-1
Zebrafish	*smo*		*(82)*	pCS2+
Mouse	$Cachd1_{ECD}$	IMAGE 6834428	Source Biosciences	pYX-Asc
Mouse	$Fzd5_{CRD}$	Synthesized clone	GenScript	pNeo_sec
Human	*FZD7*	IMAGE 4549389	Source Biosciences	pOTB7
Human	$FZD7_{CRD}$	Synthesized clone	GenScript	pNeo_sec
Human	$FZD8_{CRD}$	Synthesized clone	GenScript	pNeo_sec
Human	$LRP6_{P1E1P2E2}$	IMAGE 40125687	Source Biosciences	pHL_sec
Human	$LRP6_{P3E3P4E4}$	IMAGE 40125687	Source Biosciences	pHL_sec

This is the author's version of the work. It is posted here by permission of the AAAS for personal use, not for redistribution. The definitive version was published in *Science* on 03/05/2024, DOI: 10.1126/science.ade6970.

Table S7. Riboprobe templates.

Gene	Vector	Resistance	Linearization	RNA Polymerase	Reference
aoc1	pCRII-TOPO	Amp, Kan	SpeI	T7	constructed
slc18a3b	pCRII-TOPO	Amp, Kan	XhoI	SP6	constructed
kiss1				T3	PCR-amplified
cachd1	pCRII-TOPO	Amp, Kan	SpeI	T7	constructed
zgc:101731	pCR-Blunt II-Topo	Kan, Zeocin	NotI	SP6	constructed
axin2	pSport 1	Amp	Asp718	SP6	*(83)*
selenop2	pBS KS+	Amp	SalI	T7	*(84)*
otx5	pBS	Amp	NotI	T7	*(8)*
kctd12.2	pBK-CMV	Kan	BamHI	T7	*(31)*
kctd8	pCRII-TOPO	Amp, Kan	XhoI	SP6	*(31)*
kctd12.1	pBK-CMV	Kan	EcoRI	T7	*(8)*
prss1	pCRII-TOPO	Amp, Kan	XhoI	SP6	*(85)*
spaw	pGEMT-EASY	Amp	SpeI	T7	*(86)*
lefty1	pBS SK+	Amp	NotI	T7	*(87)*
aldh1a3	pGEMT-EASY	Amp	SalI	T7	*(88)*
dbx1b	pCRII-TOPO	Amp, Kan	BamHI	T7	*(26)*
wnt3a	pBS	Amp	SmaI	T7	*(89)*
lef1	pCR-Blunt II-Topo	Kan, Zeocin	SacI	T7	*(90)*

This is the author's version of the work. It is posted here by permission of the AAAS for personal use, not for redistribution. The definitive version was published in *Science* on 03/05/2024, DOI: 10.1126/science.ade6970.

Table S8. HCR probe sets for zebrafish *cachd1* and *lrp6*.

Gene	Amplifier	Name	Sequence
cachd1	B1	cachd1_B1_7	TTCTTGGATTGTTGGCGCAGGGTAA
cachd1	B1	cachd1_B1_8	AACTGCGGTAAACAGACCAGCATTA
cachd1	B1	cachd1_B1_9	GCGTCAAGGCATAGTTTTAGGCACT
cachd1	B1	cachd1_B1_10	TCCGGACTCCGGTAGGGTTACTAAG
cachd1	B1	cachd1_B1_14	CTGTCCGCGACCGACTCGCCATTGT
cachd1	B1	cachd1_B1_15	TCCACGCCGCGAAGGAGAATCGAGG
cachd1	B1	cachd1_B1_44	AGATTTTATCATGTTCGTCGATGGA
cachd1	B1	cachd1_B1_45	GAACAGTATCTGCTATCGTCAACAC
cachd1	B1	cachd1_B1_65	TGAAGGTGGCCACGTCGATCATTCG
cachd1	B1	cachd1_B1_66	CTCCCATCTGGTCCGCATAAGGCAA
cachd1	B1	cachd1_B1_82	CATAAACTATGCAGAGGATAAACGA
cachd1	B1	cachd1_B1_83	GCTGCTTCACCGGGATCTCTGGCTG
cachd1	B1	cachd1_B1_86	GAAGGCAGGAGCTGGGCTGCCCCAG
cachd1	B1	cachd1_B1_87	TCTCGACTGTAGCGAGCTGTTTAAA
cachd1	B1	cachd1_B1_88	TACCAGCGGACAACATCACCGTAGG
cachd1	B1	cachd1_B1_89	TCAGGTGCTCATAGGGAGAGGAGAA
cachd1	B1	cachd1_B1_91	TGTCGCTCAGGTAAGCAGTGTAGTG
cachd1	B1	cachd1_B1_92	GGCCGGGGTTGGCTATAAGTCGAGT
cachd1	B1	cachd1_B1_93	TCACCTCATTCCTCACAGAAGACTT
cachd1	B1	cachd1_B1_94	ATTCATCAGTCACGTGGCTGGTGGC
cachd1	B1	cachd1_B1_96	TGTAACGCCTCACAATGTAGCAGTT
cachd1	B1	cachd1_B1_97	TCCGCAGCACCCCATTGGGCGTTGC
cachd1	B1	cachd1_B1_98	CTTTGTCCATGAGTGAACCGGGGTA
cachd1	B1	cachd1_B1_99	ACCATTGCCTCCTGGTGGGATCGAA
cachd1	B1	cachd1_B1_103	TAGGGGCGTGGATGGTGTGGCTAAT
cachd1	B1	cachd1_B1_104	CTGTGTAACCAGAGGCCATTTGGGA
cachd1	B1	cachd1_B1_109	GGGCCACCAAATAGCCTCTGTCCTC

This is the author's version of the work. It is posted here by permission of the AAAS for personal use, not for redistribution. The definitive version was published in *Science* on 03/05/2024, DOI: 10.1126/science.ade6970.

cachd1	B1	cachd1_B1_110	GACCCTTCGGATCAATCAGTGTCGG
cachd1	B1	cachd1_B1_120	AGAAGGCCAGCGCATCACACGTCTC
cachd1	B1	cachd1_B1_121	AGAGACGGTCCACAGTACTGCAAGC
cachd1	B1	cachd1_B1_126	AAGGCTCCTGGTGCACGTCACAGCT
cachd1	B1	cachd1_B1_127	GACTGGGCTCAATTACAGTCAAAGA
cachd1	B1	cachd1_B1_134	CATAAGGACTCTTGGCGCCCACTAT
cachd1	B1	cachd1_B1_135	CCTCATCTAAAATGCCCATTCCATC
cachd1	B1	cachd1_B1_139	GGTGCCTATAGGCATAAACTGCCAA
cachd1	B1	cachd1_B1_140	TGTGCTGATGACTGCGACGGTGGAT
cachd1	B1	cachd1_B1_150	CCGCTGAGAGAGGATCGTTATTGCA
cachd1	B1	cachd1_B1_151	CCTCGTCGTGATTGCCCACATCAAC
cachd1	B1	cachd1_B1_168	ACCTTTACGGGCCTGAGAAAAGCTG
cachd1	B1	cachd1_B1_169	ACAAAACCAGGGCTGGGTACTAAAA
lrp6	B5	lrp6_B5_9	TAAACAGCGTCCGTTTAATAGACTC
lrp6	B5	lrp6_B5_10	TCTGAACGCCGCTGGGCGCCGAGCC
lrp6	B5	lrp6_B5_20	CCACGATGACAGAGCGCAGCGACCC
lrp6	B5	lrp6_B5_21	GGCCGTTGGGCCAGTAGATCTCCGT
lrp6	B5	lrp6_B5_31	GCTGGCTGTAGACGTGGATGTCCAT
lrp6	B5	lrp6_B5_32	GGCTCGCCACGTCCATGGGCTGGCG
lrp6	B5	lrp6_B5_35	TTGGACAGGCGCACTGATAGTAGGG
lrp6	B5	lrp6_B5_36	TGTGGTCCTCCAGCAGCTGTACGCC
lrp6	B5	lrp6_B5_38	CTGTGCGGCGCGCTAGCAGGAGGAG
lrp6	B5	lrp6_B5_39	GCGTGTCCAGAGAGATCCGGCGCAG
lrp6	B5	lrp6_B5_45	GCGAGGTCACCACCAGCTGAGCGTC
lrp6	B5	lrp6_B5_46	CGGCGATGCCGTCCGGGTGGTTCAC
lrp6	B5	lrp6_B5_66	TCACCCCGAAGGTCTGGTGGACGAA
lrp6	B5	lrp6_B5_67	AGCCGCCATTAGCCCACGCACACGG
lrp6	B5	lrp6_B5_87	CCATCGCTGCACGGTCTATCTTGGG
lrp6	B5	lrp6_B5_88	GCACCAGAGTGATGCGGCCCGACCC

This is the author's version of the work. It is posted here by permission of the AAAS for personal use, not for redistribution. The definitive version was published in *Science* on 03/05/2024, DOI: 10.1126/science.ade6970.

lrp6	B5	lrp6_B5_113	CGAGGTTTGAGCCGCCAACAGCGCC
lrp6	B5	lrp6_B5_114	CGATGCTCAGGTCGTACGGCTGCAG
lrp6	B5	lrp6_B5_126	CGCTGCTCTCGATGCGCCGCAGGTC
lrp6	B5	lrp6_B5_127	TCACAATCCGATTGGCTCCGGACAG
lrp6	B5	lrp6_B5_132	GCGCCTGTATTTTGGTTCGTCCCTC
lrp6	B5	lrp6_B5_133	CGTGGATGTCGCTCAGTGAGGCGAT
lrp6	B5	lrp6_B5_141	GGATACAGTCCACCTCACCCGACAC
lrp6	B5	lrp6_B5_142	CAAACCCGTCACAGCGCCACGCCTG
lrp6	B5	lrp6_B5_147	CGTCGGAGCGGTCCTGGCAGTTGAT
lrp6	B5	lrp6_B5_148	CAGGGCACAGAACTTCACACTTGTT
lrp6	B5	lrp6_B5_152	CTGTCGCATAGCAGCCGATCTCGTC
lrp6	B5	lrp6_B5_153	TGTTAGTGGGAGCAAACGACGGCTC
lrp6	B5	lrp6_B5_155	ACACCGCGCCGACCACGAACAGCAC
lrp6	B5	lrp6_B5_156	GGCAGAGCACGCGCTGGCACACGAA
lrp6	B5	lrp6_B5_158	GTCCGTGAACCACGAAGTCATTGGT
lrp6	B5	lrp6_B5_159	GGACGTATCCCAGCGGCACCGGCGG
lrp6	B5	lrp6_B5_162	CTCCCATGATGCTCAGCGAGCCCAT
lrp6	B5	lrp6_B5_163	CGCGGTCGTACGGTGGTCCACTGCT
lrp6	B5	lrp6_B5_170	CGAAGTGGCGGTAACTGTACGGCCG
lrp6	B5	lrp6_B5_171	CCGTGCTGCACGGCGTCGTCGGAGG
lrp6	B5	lrp6_B5_175	GCAGCGGCTCCGAGTCGTAGTTCAG
lrp6	B5	lrp6_B5_176	ACTGGCTGCGCGGCGTGGGCGGCGG
lrp6	B5	lrp6_B5_178	GCTCGGTGTACGGTGACGGCGGGCA
lrp6	B5	lrp6_B5_179	GCGGGTACAGCTGGTGCGAGTAGCT
